# Development‐Informed Microsphere Assemblies Enable Spatiotemporally Programmed Bone–Vascular Organoid‐Like Constructs for Large Bone Defect Repair

**DOI:** 10.1002/advs.78616

**Published:** 2026-11-09

**Authors:** Renhao Ni, Xinyi Shi, Tong Zhu, Guanrong Li, Lei Chang, Shengtao Li, Jingyi Chi, Chen Shi, Zeming Hu, Rong Xu, Yingchun Zhu, Yang Luo, Yichen Chen, Fan Yang, Yabin Zhu

**Affiliations:** ^1^ Health Science Center Ningbo University Ningbo China; ^2^ Department of Orthopaedics Shanghai Key Laboratory for Prevention and Treatment of Bone and Joint Diseases Shanghai Institute of Traumatology and Orthopaedics, Ruijin Hospital Shanghai Jiao Tong University School of Medicine Shanghai China; ^3^ The First Affiliated Hospital of Ningbo University Ningbo China; ^4^ Chinese Academy of Medical Sciences & Peking Union Medical College Beijing China; ^5^ State Key Laboratory of Experimental Hematology National Clinical Research Center for Blood Disease Haihe Laboratory of Cell Ecosystem Institute of Hematology & Blood Diseases Hospital Tianjin China; ^6^ Central Laboratory Women and Children's Hospital of Ningbo University Ningbo China

**Keywords:** bone–vascular organoid‐like constructs, development‐informed gene programming, large segmental bone defects, matrigel‐free assembled microsphere, osteogenesis–angiogenesis coupling

## Abstract

Large segmental bone defects are difficult to repair because endogenous stem‐cell activation, vascular ingrowth, and osteogenic maturation are insufficient to rebuild organized bone tissue. Bone organoids offer a promising regenerative strategy, but current systems are limited by Matrigel dependence, poor vascularization, low post‐implantation survival, and inadequate reconstruction of hierarchical bone microarchitecture. Here, we report a development‐informed, Matrigel‐free bone–vascular organoid‐like platform that integrates single‐cell‐guided gene programming with an osteoinductive and vascular‐permissive microsphere matrix. Single‐cell analysis identified *BMP4* and *VEGFA* as key genes linking osteogenic commitment with vascular morphogenesis. Engineered BMSCs were used to sequentially generate osteoinductive bone organoid‐like constructs(OLCs) and bone–vascular OLCs with coordinated osteogenic and angiogenic activity. Microfluidic GelMA/PEO/bioactive glass(BG) microsphere clusters provided adhesive, mineralization‐promoting, and space‐making cues for cell migration, matrix deposition, and endothelial invasion. Within seven days, the OLCs developed mineralized matrix, vascular‐like sprouting, and early vascular‐like microchannels. After implantation into rabbit femoral defects, they enhanced vascularized bone formation, defect bridging, and trabecular remodeling. This work demonstrates the power of development‐informed, Matrigel‐free bone–vascular OLCs in accelerating vascularized bone regeneration for large segmental bone defect repair.

## Graphical abstract

Single‐cell‐guided prioritization of BMP4 and VEGFA is combined with temporally programmed BMSCs and a Matrigel‐free GelMA/PEO/bioactive‐glass assembled‐microsphere matrix to generate bone–vascular organoid‐like constructs. The platform coordinates osteogenic differentiation, endothelial activation, vascular‐like microchannel formation, and vascularized bone regeneration through temporally programmed BMP4 and VEGFA overexpression, providing a development‐informed strategy for large bone defect repair.

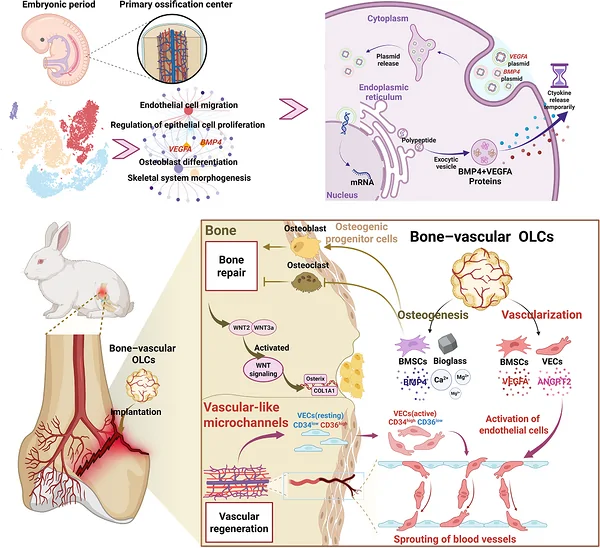

## 1 Introduction

With global population aging and the rising incidence of severe trauma, tumor resection, infection‐related debridement, osteoporosis, and delayed fracture healing, large segmental bone defects have become a major clinical challenge [1]. These defects often exceed the intrinsic regenerative capacity of bone and are accompanied by insufficient vascularization, impaired osteogenesis, delayed union, or non‐union [2]. Current clinical options remain inadequate. Metallic and polymeric implants provide mechanical support but lack biological activity and vascular integration, compromising long‐term osseointegration [3]. Autografts remain the clinical gold standard but are limited by donor‐site morbidity and finite tissue availability, whereas allografts and recombinant osteoinductive factors carry risks of immune rejection, pathogen transmission, poorly controlled spatiotemporal exposure, and ectopic ossification [4]. These limitations highlight the need for biologically active, vascularized, and structurally organized living grafts capable of accelerating large bone defect repair [5].

A central obstacle in engineering such grafts is the difficulty of directing stem‐cell fate within a defined spatial and temporal microenvironment [6]. Bone regeneration is not driven by osteogenic differentiation alone, but requires coordinated interactions among mesenchymal stromal cells, osteogenic lineage cells, endothelial cells, immune components, and extracellular‐matrix niches [7]. During skeletal development, these populations communicate within organized microenvironments to synchronize osteogenesis, angiogenesis, matrix deposition, and mineralization [8]. In adult segmental defects, however, this regenerative niche is disrupted, leading to insufficient endogenous activation of bone marrow mesenchymal stromal cells and poor coupling between vascular ingrowth and bone formation [9]. Identifying the developmental signals and niche features that govern skeletal lineage commitment is therefore essential for constructing engineered bone tissues with predictable differentiation and regenerative function [10].

Bone organoids provide a promising strategy for reconstructing key aspects of skeletal development and regeneration in vitro [11]. As self‐organizing 3D cellular systems, they can capture multicellular interactions, lineage specification, and tissue‐like maturation more effectively than conventional 2D cultures [12]. However, current bone organoid systems remain constrained by several bottlenecks [13]. Many protocols rely on basement‐membrane extracts such as Matrigel, whose undefined composition and batch‐to‐batch variability limit reproducibility, mechanistic interpretation, and translational scalability [14]. In addition, the lack of a functional vascular network restricts oxygen and nutrient exchange, resulting in central necrosis, fibrosis, limited organoid size, and poor post‐implantation survival [15]. Existing constructs also frequently achieve only early osteogenic induction, but fail to sustain long‐term lineage‐coupled maturation or promotes formation of organized bone‐like microarchitecture, particularly osteon‐like structures and vascular‐like microchannels [16]. Thus, next‐generation bone organoids require not only instructive cellular programs, but also a defined, vascular‐permissive, and osteoinductive matrix that can support tissue maturation and in vivo integration [17].

For bone organoids intended for implantation, the matrix should not merely maintain 3D cell aggregation, but should actively instruct osteogenesis while permitting vascular infiltration and tissue‐scale remodeling [1]. This requirement is difficult to meet using conventional Matrigel‐based systems, whose undefined biochemical composition, tumor‐derived origin, and batch‐to‐batch variability compromise mechanistic clarity, reproducibility, and translational compatibility [18]. We therefore designed a Matrigel‐free assembled‐microsphere matrix based on the complementary functions of GelMA, bioactive glass, and PEO. GelMA served as a cell‐adhesive hydrogel backbone, enabling microfluidic generation of size‐controlled microspheres and supporting cell attachment within the organoid niche [19]. Bioactive glass was introduced to compensate for the limited osteoinductive capacity of GelMA by releasing bone‐regenerative ions and providing mineralization cues that favor osteogenic differentiation and extracellular‐matrix maturation [20]. PEO was further incorporated as a transient phase‐separating component [21]. During microsphere formation, liquid–liquid phase separation between PEO‐rich domains and the GelMA‐rich network generated micron‐scale voids after PEO leaching, thereby creating vascular‐permissive microcavities for nutrient transport, cell migration, matrix deposition, and endothelial invasion.

To further guide cellular organization within this material niche, we reasoned that skeletal development could serve as a biological blueprint for repair‐oriented bone organoid engineering [22]. Using single‐cell analysis of clinical specimens and developmental skeletal tissues, we examined the cellular ecology of bone regeneration and identified insufficient endogenous osteogenic activation of BMSCs as a key feature of severe segmental non‐union. In contrast, embryonic skeletal development displays rapid and coordinated bone formation, suggesting that developmental stem‐cell niches contain regenerative programs that may be reactivated for adult bone repair. By comparing these cellular states, we identified *BMP4* and *VEGFA* as key effector signals coupling osteogenic lineage commitment with vascular sprouting, providing the molecular basis for stepwise integration of lineage induction, vascularization, and structural maturation.

Here, we establish a spatiotemporally programmed bone–vascular organoid‐like construct system for large segmental bone defect repair by coupling development‐informed gene programming with a Matrigel‐free assembled‐microsphere matrix (Figure 1). Guided by single‐cell analysis of skeletal regenerative niches, BMSC^BMP4+VEGFA^ were introduced to provide localized paracrine cues for coordinated osteogenic commitment and vascular morphogenesis. Meanwhile, a microfluidic GelMA/BG/PEO microsphere scaffold was designed as a defined, osteoinductive, and vascular‐permissive niche that supports cell migration, nutrient diffusion, extracellular‐matrix remodeling, and tissue infiltration. Within this engineered microenvironment, the bone–vascular organoid‐like constructs developed organized collagen deposition, mineralized matrix formation, vascular‐like sprouting, and early vascular‐like microarchitectures within seven days. Following implantation into rabbit femoral defects, the matured OLCs survived in the marrow cavity, enhanced vascularized bone formation, promoted defect bridging, and supported trabecular remodeling. Collectively, this work presents a development‐informed and Matrigel‐free vascularized bone OLCs platform that integrates instructive cellular programming with an osteoinductive and space‐making material niche for the repair of large segmental bone defects.

**FIGURE 1:**
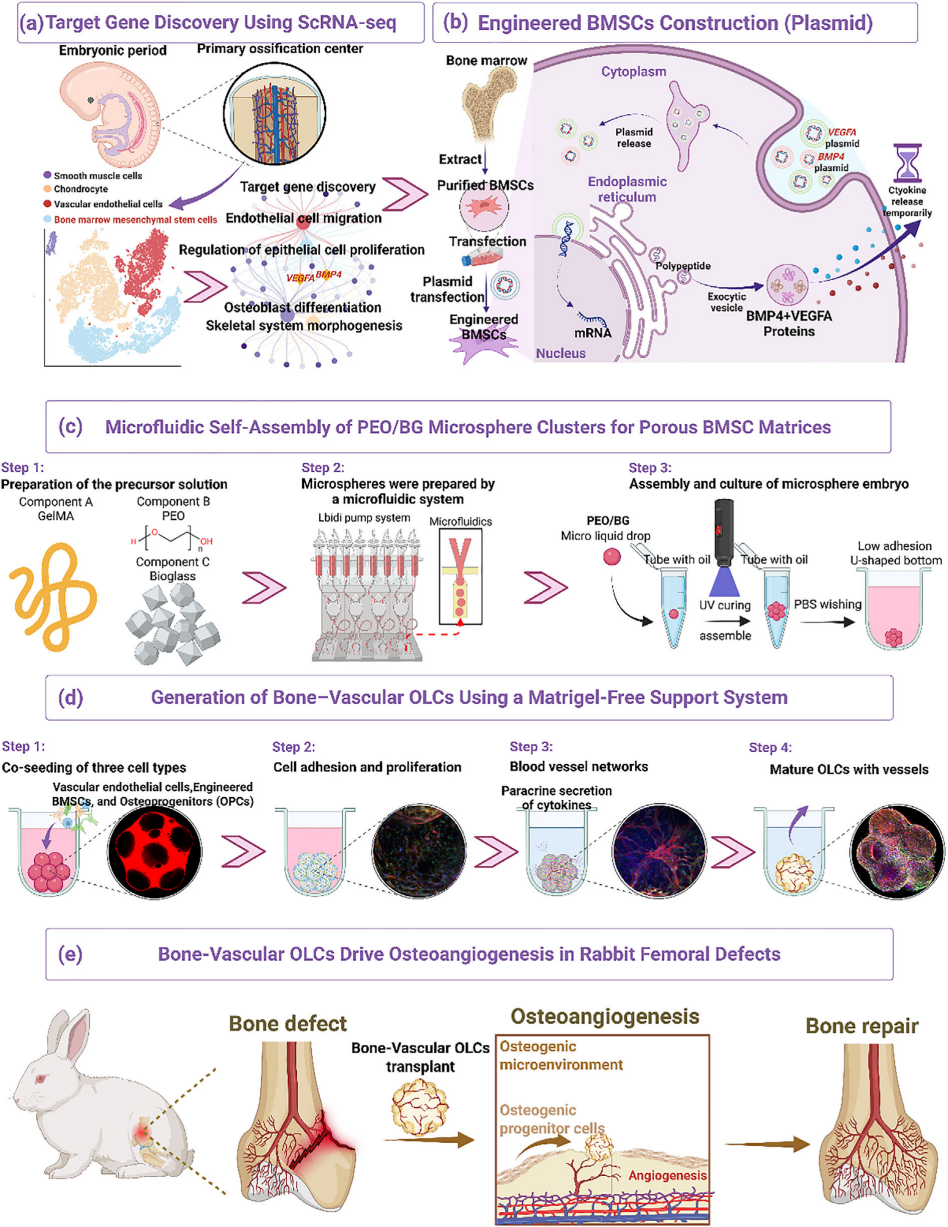
Schematic of development‐informed bone–vascular OLCs engineering strategy for large bone defect repair. (a) Single‐cell RNA sequencing‐guided target discovery from embryonic skeletal datasets. Major skeletal developmental cell populations, including stromal mesenchymal cells, chondrocytes, vascular endothelial cells, and bone marrow mesenchymal stem cells, are identified, followed by cell–cell interaction analysis to screen osteogenesis–angiogenesis‐associated paracrine cues. ***BMP*** *4* and ***VEGFA*** are selected as key developmental targets for engineering bone–vascular OLCs. (b) Engineering of BMSCs with osteogenic and angiogenic paracrine activity. BMSCs are transiently transfected with plasmids, enabling intracellular gene expression, protein synthesis, vesicular trafficking, and sustained cytokine secretion to provide temporally programmed osteogenic and vascular cues. (c) Microfluidic self‐assembly of porous PEO/BG microsphere clusters. GelMA precursor solutions containing PEO and bioactive glass are emulsified into droplets, which undergo PEO‐mediated clustering and photocrosslinking to form porous assembled microspheres with a cell‐permissive architecture and ion‐releasing bioactivity. (d) Matrigel‐free generation of self‐organized bone–vascular OLCs. Engineered BMSCs, vascular endothelial cells, and osteoprogenitor cells are co‐seeded onto PEO/BG assembled microspheres in ultra‐low‐adhesion culture, where the porous matrix supports multicellular aggregation, migration, extracellular matrix deposition, endothelial network formation, and osteogenic maturation. (e) In vivo repair mechanism in a rabbit critical‐sized femoral defect model. Implanted bone–vascular OLCs act as living osteoangiogenic units that integrate engineered BMP4/VEGFA paracrine signaling, BG‐derived ionic stimulation, and porous microsphere‐guided tissue organization. These synergistic cues recruit host osteogenic and endothelial cells, activate osteogenic signaling, promote vascular sprouting, and accelerate coupled osteogenesis–angiogenesis, vascular‐like microchannels, and large bone defect repair.

## 2 Results

### 2.1 Fabrication of Bone–Vascular Organoid‐Like Constructs for Rapid Repair of Large Bone Defects

Severe segmental bone defects remain a major clinical challenge and frequently progress to delayed union or non‐union, partly because endogenous bone marrow mesenchymal stromal cells (BMSCs) are insufficient to initiate a robust and spatially coordinated osteogenic regenerative response. In contrast, embryonic skeletogenesis proceeds through rapid, highly organized, and tightly coupled osteogenesis and angiogenesis, suggesting that developmental skeletal environments provide a reference framework for identifying osteogenic–angiogenic coupling signals relevant to bone regeneration [23]. To capture these pro‐regenerative signals, we adopted a single‐cell transcriptomics‐guided strategy to interrogate early skeletal developmental datasets and identify candidate paracrine programs associated with osteogenic–angiogenic coupling.

Single‐cell RNA sequencing analysis of embryonic skeletal tissues resolved major developmental cell populations, including stromal mesenchymal cells, chondrocytes, vascular endothelial cells, and BMSC‐like progenitor populations. Subsequent cell–cell interaction analysis highlighted *BMP4* and *VEGFA* as key developmental cues associated with early osteogenesis and vascular formation (Figure 1a). Because sustained and non‐physiological secretion of BMP4 or VEGFA may increase the risks of heterotopic ossification and aberrant neovascularization, we avoided stable viral overexpression and instead used transient plasmid transfection to engineer BMSCs. BMSCs were co‐transfected with BMP4‐ and VEGFA‐encoding plasmids to generate a temporally programmed paracrine profile characterized by high early secretion followed by gradual decline. This early‐high and late‐low release pattern was confirmed by ELISA analysis of BMP4 and VEGFA secretion over time, supporting transient transfection as a controllable strategy for delivering developmental cues during the early phase of OLCs maturation (Figure 1b). After cellular uptake, plasmid DNA was transcribed into mRNA, followed by protein translation and processing through the endoplasmic reticulum–Golgi secretory pathway, enabling time‐dependent BMP4/VEGFA secretion while reducing the risk of long‐term aberrant signaling.

To provide engineered BMSCs with a stable, controllable, and Matrigel‐independent 3D niche, we developed a porous microsphere assembly platform based on microfluidic droplet emulsification and in situ photocrosslinking. A photocrosslinkable GelMA precursor containing poly(ethylene oxide) (PEO) and 1393 bioactive glass (BG) was emulsified using a microfluidic device to generate monodisperse droplets, which were collected into a 1.5 mL tube prefilled with mineral oil (Figure 1c). Through PEO‐mediated bridging flocculation, adjacent droplets spontaneously assembled into embryoid body‐like clusters in the oil phase before being crosslinked in situ under 405 nm irradiation. After crosslinking, the constructs were washed with PBS to remove residual oil and transferred to ultra‐low‐attachment U‐bottom plates for subsequent culture. Within the assembled microspheres, PEO played dual structural roles. First, it promoted mesoscale droplet clustering through inter‐droplet bridging before photocrosslinking. Second, PEO‐driven liquid–liquid phase separation and subsequent leaching generated a honeycomb‐like porous architecture within the microsphere assemblies. In parallel, BG provided osteoinductive ionic cues through sustained low‐dose release of Ca^2^^+^ and Mg^2^^+^. Thus, PEO and BG performed complementary functions, in which PEO programmed microsphere assembly and pore formation, whereas BG supplied bioactive ionic stimulation that biased cells toward an osteogenic phenotype. The resulting assembled microspheres (aMS) formed an interconnected and highly porous network that supported nutrient diffusion, cell migration, cell–cell interaction, and extracellular matrix deposition.

Bone–vascular OLCs were then generated by integrating engineered cells with the PEO/BG aMS platform (Figure 1d). BMP4/VEGFA‐programmed BMSCs, hereafter referred to as BMSC^BMP4+VEGFA^, were co‐seeded with vascular endothelial cells and osteoprogenitors onto PEO/BG aMS and cultured under ultra‐low‐attachment conditions. We define these constructs as bone–vascular OLCs because they combine multicellular self‐organization, endothelial network formation, osteogenic maturation, and matrix deposition within a 3D microstructured niche. The porous topology of aMS, together with intrinsic RGD motifs in GelMA, promoted cell adhesion, proliferation, and inter‐microsphere bridging, enabling cells to progressively migrate and organize along interconnected pore channels. Meanwhile, BMP4/VEGFA secretion from BMSC^BMP4+VEGFA^ provided temporally programmed osteogenic and angiogenic cues, while BG‐derived Ca^2^^+^/Mg^2^^+^ further supported osteogenic differentiation and biomineralization. This biomaterials–cell co‐design enabled Matrigel‐free formation of self‐organized bone–vascular OLCs and provided both biochemical cues, including BMP4, VEGFA, Ca^2^^+^, and Mg^2^^+^, and biophysical cues, including pore architecture and microsphere‐guided spatial organization.

To evaluate their regenerative potential, bone–vascular OLCs were implanted into a rabbit distal femoral defect model (Figure 1e). Within the defect microenvironment, the OLCs were designed to act as living osteoangiogenic units that combine engineered paracrine signaling, BG‐mediated ionic stimulation, and porous microsphere‐guided tissue organization. BMSC^BMP4+VEGFA^‐derived BMP4 and VEGFA were expected to promote osteogenic differentiation and vascular activation during the early repair phase, whereas BG‐derived ions provided additional osteoinductive stimulation. The porous aMS backbone further facilitated host cell infiltration, tissue ingrowth, and spatial coupling between nascent vascular structures and newly deposited bone matrix. Together, these synergistic cues promoted coordinated osteogenesis and angiogenesis, thereby supporting defect bridging and bone remodeling in large bone defects.

Collectively, this developmental‐informed strategy integrates single‐cell‐guided cue selection, transient gene‐programmed BMSC paracrine activity, and self‐assembled PEO/BG microsphere architecture to generate implantable bone–vascular OLCs. By combining temporally controlled biochemical signaling with a porous and osteoinductive material niche, this platform provides a tunable and biomimetic approach for modeling vascularized bone regeneration in vitro and accelerating the repair of large bone defects in vivo.

### 2.2 Singl**e‐**Cel**l** Ana**l**ysis of Skeletal Regenerative Contexts Identified *BMP4* and *VEGFA* as Candidate Regulators Associated With Osteogenesis–Angiogenesis Coupling

Severe segmental bone defects remain a major clinical challenge and frequently progress to non‐union, in part because endogenous BMSCs fail to mount a sufficiently robust osteoregenerative response. In contrast, embryonic skeletogenesis achieves rapid and highly coordinated bone formation, suggesting that early developmental BMSC programs may provide actionable cues for enhancing adult bone repair. To capture such pro‐regenerative signals, we applied an integrative bioinformatics strategy to prioritize key transcriptional programs from the early skeletal developmental niche and focused subsequent analyses on single‐cell transcriptomic profiles of the stem/progenitor microenvironment.

We integrated the scRNA‐seq dataset GSE156635, including BSC1–BSC4, yielding 18,652 cells that segregated into four major populations on the t‐SNE embedding: BMSCs, chondrocytes, endothelial cells, and smooth muscle cells (Figure 2a). Lineage annotation was supported by canonical marker expression (Figure 2b). Together, these populations represent the core mesenchymal, chondrogenic, endothelial, and perivascular components of the early skeletal niche. Differential expression analysis identified genes preferentially expressed in the BMSC cluster relative to chondrocytes, endothelial cells, and smooth muscle cells. GO enrichment analysis of these BMSC‐biased genes highlighted extracellular matrix and structural organization, skeletal‐development programs, including osteoblast differentiation and skeletal system morphogenesis, as well as vascular and migration‐related processes, including endothelial cell migration and regulation of vasculature development (Figure 2c). These results are consistent with a niche‐associated role of developmental BMSCs in coordinating osteogenesis with vasculature‐associated remodeling. Network‐based gene–function mapping using cnetplot analysis further nominated *BMP4* and *VEGFA* as highly connected candidate regulators across multiple enriched biological processes (Figure 2d). Feature plots revealed heterogeneous expression of both genes within the integrated embedding, consistent with their context‐dependent roles in the developmental microenvironment (Figure 2e). We prioritized *BMP4* and *VEGFA* because they were highly connected in the gene–function network, displayed niche‐relevant expression patterns, and represented complementary osteogenic and angiogenic regulatory axes.

**FIGURE 2:**
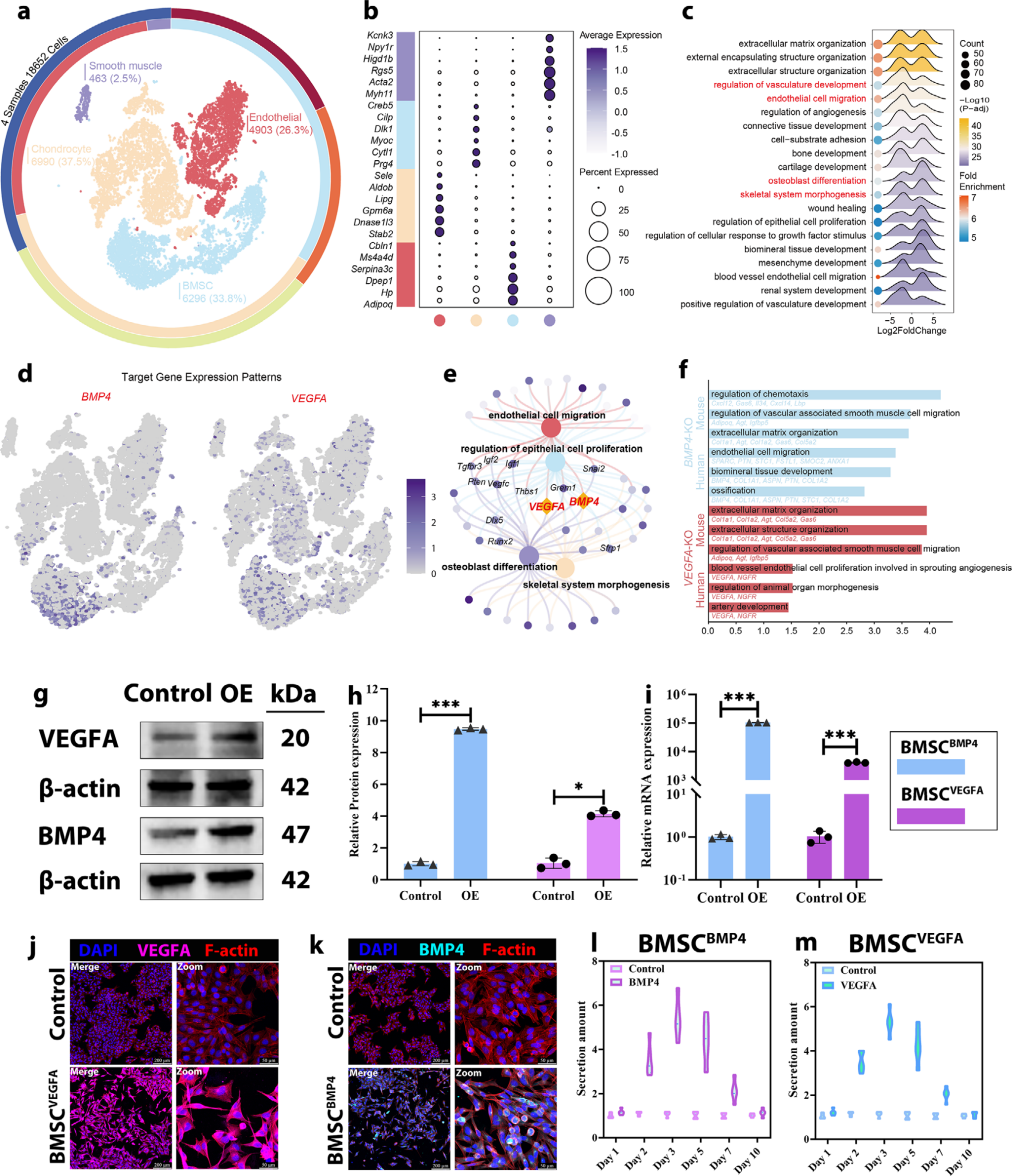
Single‐cell analysis of the skeletal developmental microenvironment prioritizes BMP4 and VEGFA and validates their in silico knockout effects, overexpression and time‐resolved secretion kinetics in BMSCs. (a) t‐SNE embedding of the integrated scRNA‐seq dataset (GSE156635; four samples, BSC1–BSC4) comprising 18,652 cells annotated into four major populations: BMSCs (6,296; 33.8%), chondrocytes (6,990; 37.5%), endothelial cells (4,903; 26.3%) and smooth muscle cells (463; 2.5%). (b) Dot plot of representative marker genes supporting annotation of the four cell types; color indicates average expression and dot size indicates the fraction of cells expressing each gene. (c) Gene Ontology (GO) biological process enrichment analysis of BMSC differentially expressed genes, highlighting extracellular matrix/structure organization and skeletal/vascular programs (including osteoblast differentiation, skeletal system morphogenesis, endothelial cell migration and regulation of vasculature development). (d) Gene–function association network (cnetplot) for selected enriched terms, identifying BMP4 and VEGFA as highly connected candidate regulators. (e) Feature plots showing BMP4 and VEGFA expression across the integrated t‐SNE embedding. (f) In silico knockout analysis: BMP4 or VEGFA was individually ablated in human and mouse BMSCs, and significantly altered genes and associated pathway/GO enrichments were assessed to evaluate the impact of BMP4/VEGFA loss on skeletal‐ and vasculature‐related molecular programs. (g) Representative immunoblots of VEGFA and BMP4 in wild‐type/vector control (WT/control) and overexpression (OE) BMSCs; β‐actin was used as a loading control. (h) Densitometric quantification of immunoblot bands in g (n = 3 independent experiments). (i) qRT–PCR quantification of BMP4 and VEGFA mRNA in control versus OE BMSCs (n = 3 independent experiments). (j, k) Immunofluorescence staining of VEGFA (magenta) and BMP4 (cyan) with nuclei (DAPI, blue) and F‐actin (phalloidin, red); merged images and magnified views are shown for control/blank cells (j) and OE cells (k). (l, m) ELISA time course of secreted BMP4 (l, BMSC^BMP4^) and VEGFA (m, BMSC^VEGFA^) in culture supernatants collected on days 1–10. Data are mean ± s.d. (n = 3 independent experiments). Statistical significance: *p < 0.05, ** *p* < 0.01, *** *p* < 0.001 (two‐tailed tests). Scale bars as indicated.

To further infer the functional relevance of these candidates in osteogenic–angiogenic coupling, we performed in silico knockout of *BMP4/Bmp4* or *VEGFA/Vegfa* in a cross‐species setting using human and mouse BMSCs and assessed the resulting gene‐level changes and functional enrichments (Figure 2f and Figure S1). In mouse BMSCs, virtual ablation of *Bmp4* or *Vegfa* generated perturbation‐associated gene signatures ranked by Z score (Figure S1a,b), indicating that these axes engage distinct yet complementary molecular modules. GO enrichment analyses suggested that Bmp4 loss preferentially affected migration and niche‐interaction programs, including chemotaxis, endothelial cell migration, and extracellular matrix organization, while also influencing ossification‐related terms, including endochondral ossification, and biomineralization‐associated processes (Figure S1c). By contrast, *Vegfa* ablation more prominently altered vasculature‐related programs, including regulation of vasculature development and vascular cell migration/proliferation linked to sprouting angiogenesis, together with marked changes in extracellular matrix and structural organization (Figure S1c). Applying the same in silico knockout framework to human BMSCs from the independent GSE143753 dataset yielded highly concordant functional enrichment patterns. Loss of *BMP4* or *VEGFA* converged on a coupled system involving extracellular matrix remodeling, tissue morphogenesis, osteogenic and mineralization programs, and vasculature‐associated processes (Figure S1d). This cross‐species concordance reduces the likelihood of species‐ or dataset‐specific bias and strengthens the robustness of our inference that *BMP4* and *VEGFA* may act as key regulatory nodes linking osteogenesis and angiogenesis in the early developmental microenvironment. On the basis of this evidence (Figure 2f and Figure S1a–d), we selected *BMP4* and *VEGFA* as actionable development‐informed factors for engineering adult BMSCs to amplify early reparative programs.

To further define BMSC‐associated transcriptional programs involved in bone‐vessel coupling, we performed hdWGCNA on the annotated single‐cell dataset. Hierarchical clustering of co‐expressed genes identified multiple modules, including black, red, green, blue, turquoise, brown, and yellow modules (Figure S13a). Module‐trait correlation analysis revealed distinct cell‐type associations among these modules. Notably, the blue module showed the strongest positive association with BMSCs, whereas the turquoise module was preferentially associated with endothelial cells and the red/brown modules were associated with chondrocytes (Figure S13b). Therefore, the blue module was selected as the BMSC‐associated module for downstream candidate prioritization. The module UMAP further demonstrated a clear separation of gene modules in the co‐expression network space, supporting the modular organization of the hdWGCNA network (Figure S13c). BMP4 and VEGFA were located within the BMSC‐associated blue module region, suggesting that both genes were embedded in a BMSC‐related co‐expression program. Consistently, module eigengene projection on the t‐SNE embedding showed that the blue module activity was enriched in the BMSC population, whereas the turquoise module was enriched in endothelial cells and other modules showed distinct cell‐type‐biased activity patterns (Figure S13d).

We next evaluated intercellular communication among the major cell populations using CellChat. Both the number and strength of inferred ligand‐receptor interactions indicated active communication between BMSCs and endothelial cells (Figure S13e), supporting the relevance of focusing on BMSC‐to‐endothelial signaling in subsequent candidate screening. To prioritize osteogenic and angiogenic BMSC‐derived candidate ligands, we intersected BMSC upregulated DEGs, genes from the BMSC‐associated blue module, and function‐specific GO gene sets. For the osteogenic axis, the bone mineralization GO term was used. This intersection identified 11 candidate genes (Figure S13f). Among candidates with available BMSC‐to‐endothelial communication evidence from CellChat and NicheNet, BMP4 showed the highest integrated ligand‐prioritization score, supporting BMP4 as the leading osteogenic‐axis candidate ligand.

For the angiogenic/endothelial axis, the endothelial cell differentiation GO term was used. The intersection of BMSC upregulated DEGs, blue module genes, and endothelial cell differentiation‐associated genes identified 4 candidate genes (Figure S13g). Integrated CellChat‐NicheNet prioritization further ranked VEGFA as the top candidate, followed by BMP4. Together, these results indicate that BMP4 and VEGFA are BMSC‐derived candidate ligands supported by differential expression, BMSC‐associated co‐expression module membership, functional GO annotation, and BMSC‐to‐endothelial communication evidence. These findings nominate BMP4 and VEGFA as potential mediators linking BMSC osteogenic programs with endothelial regulation during bone‐vessel coupling.

To experimentally validate the candidates prioritized by the computational analyses, siRNA‐mediated knockdown of *BMP4* and *VEGFA* was performed in BMSCs. *BMP4* knockdown significantly reduced the expression of *BMP2*, *COL1A1*, *RUNX2*, and *SMAD5*, whereas *VEGFA* knockdown markedly decreased the expression of *FGFR2*, *KDR*, and *VCAM1*(Figure S14). These experimental loss‐of‐function results were broadly consistent with the preceding in silico knockout analyses, which predicted that disruption of *BMP4* and *VEGFA* would impair osteogenesis‐ and vasculature‐associated molecular programs, respectively.

To evaluate whether these development‐associated signals could be introduced into BMSCs while mitigating the potential adverse consequences of sustained osteogenic or angiogenic stimulation, we generated *BMP4* and *VEGFA* overexpression plasmids and transfected purified BMSCs in vitro. Immunoblotting confirmed increased BMP4 and VEGFA protein abundance in BMP4/VEGFA‐transfected BMSCs compared with WT and vector controls (Figure 2g), which was further supported by densitometric quantification (Figure 2h). Quantitative RT–PCR validated robust upregulation of BMP4 and VEGFA transcripts following plasmid transfection (Figure 2i). Immunofluorescence imaging showed markedly stronger intracellular VEGFA and BMP4 signals in engineered BMSCs relative to blank controls, with cytoskeletal architecture visualized by phalloidin staining (Figure 2j,k).

To distinguish the individual and combined contributions of BMP4 and VEGFA, BMSCs were subjected to different combinations of BMP4 or VEGFA knockdown (KD) and overexpression (OE), followed by immunofluorescence assessment of the osteogenesis‐associated marker BMP2 and the vascular‐associated signaling marker PDGFB (Figure S11). BMP2 expression was primarily dependent on BMP4 status: BMP4 overexpression markedly increased BMP2 fluorescence under both VEGFA‐KD and VEGFA‐OE conditions, whereas VEGFA overexpression alone did not compensate for BMP4 knockdown. Notably, the BMP4/VEGFA dual‐overexpression group exhibited the highest BMP2 fluorescence, which was significantly greater than that observed in the BMP4‐OE/VEGFA‐KD group. In contrast, PDGFB expression was predominantly associated with VEGFA status. VEGFA overexpression significantly increased PDGFB fluorescence regardless of BMP4 expression, whereas BMP4 overexpression alone did not significantly alter PDGFB levels. PDGFB fluorescence was comparable between the BMP4‐KD/VEGFA‐OE and dual‐overexpression groups, indicating that the additional effect of BMP4 on this vascular‐associated marker was limited. Together, these results suggest that BMP4 mainly promotes the osteogenic‐associated BMP2 response, whereas VEGFA primarily enhances PDGFB‐associated vascular signaling. Combined BMP4/VEGFA overexpression therefore supports the simultaneous establishment of osteogenic‐ and vascular‐associated phenotypes.

Transient plasmid transfection was deliberately selected instead of lentiviral or stable overexpression systems because the intended therapeutic function was to provide an early, temporally restricted pulse of osteogenic and angiogenic signals rather than constitutive long‐term stimulation. Although integrating lentiviral vectors provide efficient and durable genetic modification and can maintain transgene expression in MSCs for several weeks, such persistence may be undesirable for potent developmental regulators such as *BMP4* and *VEGFA*. In contrast, plasmid‐mediated gene delivery does not rely on deliberate genomic integration, and plasmid copies are progressively degraded or diluted during cell proliferation, resulting in a self‐limiting expression profile [24]. This transient strategy therefore potentially reduces integration‐related safety concerns and allows the duration of BMP4/VEGFA activity to more closely match the early biological requirements of bone repair [8, 25]. To further evaluate the short‐term cytocompatibility of this engineering strategy, we assessed apoptosis and inflammatory responses in plasmid‐transfected BMSCs. Annexin V‐FITC/PI flow cytometry showed that the overall proportions of apoptotic cells remained relatively low following BMP4, VEGFA, or combined BMP4/VEGFA overexpression (Figure S12a). Moreover, qRT‐PCR analysis revealed no significant increases in the inflammatory cytokines TNF‐α or IL‐1β compared with the blank control (Figure S12b,c). These findings indicate that transient BMP4/VEGFA plasmid transfection did not induce overt inflammatory activation and exhibited acceptable short‐term cytocompatibility under the present experimental conditions.

Importantly, because prolonged BMP4/VEGFA exposure beyond the early repair phase may lead to dysregulated osteogenic or angiogenic signaling, we sought to establish a delivery mode characterized by high expression during the early reparative window followed by progressive decline. ELISA time‐course measurements showed that plasmid‐mediated BMP4 and VEGFA secretion increased rapidly, reached maximal levels approximately 3–5 days after transfection, and began to decrease from around day 7, while lower levels remained detectable through day 10 (Figure 2l,m). Based on this in vitro secretion profile and the timing of construct implantation, BMP4 and VEGFA activity after implantation would be expected to be concentrated primarily within approximately the first postoperative week. This early‐high and late‐low kinetic profile is biologically aligned with the initial phase of bone repair. Endogenous BMP4 has been reported to exhibit transient and localized expression within proliferating periosteal and callus‐forming tissues during the first several days after fracture, before overt cartilage and bone formation [26]. Meanwhile, osteoblast‐lineage‐derived VEGFA contributes to the early angiogenic response during the inflammatory phase, and VEGFA produced by early osteolineage cells is required for rapid periosteal vascularization and bone formation. Therefore, the 3–5 day peak in BMP4 and VEGFA secretion may support early osteogenic priming, endothelial recruitment, vascular sprouting, and the rapid establishment of an immature vascular network within the bone–vascular OLCs. The subsequent decline after approximately 7 days may allow the constructs to transition from signal‐driven initiation toward matrix maturation and tissue remodeling while potentially limiting the adverse effects associated with persistent BMP4/VEGFA exposure. Therefore, transient plasmid engineering provides a temporally controllable strategy for delivering developmental osteoangiogenic cues during the initial organoid‐forming and bone‐repair phases. The progressive loss of plasmid expression, together with cellular proliferation and subsequent tissue remodeling after implantation, is expected to restrict prolonged exposure to these potent paracrine factors.

Collectively, these analyses identified BMP4 and VEGFA as development‐informed regulators associated with the coupling of osteogenic and vascular programs across species. The temporally restricted expression profile of BMP4/VEGFA‐engineered BMSCs further supports their use as programmable paracrine units that deliver an early osteoangiogenic stimulus to accelerate bone–vascular OLCs maturation and promote large bone‐defect repair.

### 2.3 PEO/BG aMS Scaffolds Provide a Porous 3D Niche that Sustains Long‐Term BMSC Survival

To create a 3D niche with embryoid‐like morphology and enhanced exchange interfaces, we established a microfluidic self‐assembly strategy to fabricate PEO/BG assembled microspheres (aMS) (Figure 3a,b). Bright‐field and rhodamine fluorescence imaging confirmed the formation of spherical, uniformly crosslinked mono‐microspheres (mMS), with homogeneous fluorescence throughout the microsphere body (Figure 3a). After assembly, individual mMS packed into larger aMS aggregates with evident interstitial voids and a porous, cluster‐like architecture (Figure 3b). Confocal Z‐stack maximum‐intensity projections acquired at 20 µm intervals further verified continuous fluorescence within both mMS and aMS, indicating effective photocrosslinking and a spatially interconnected geometry that is favorable for nutrient transport and cell infiltration.

**FIGURE 3:**
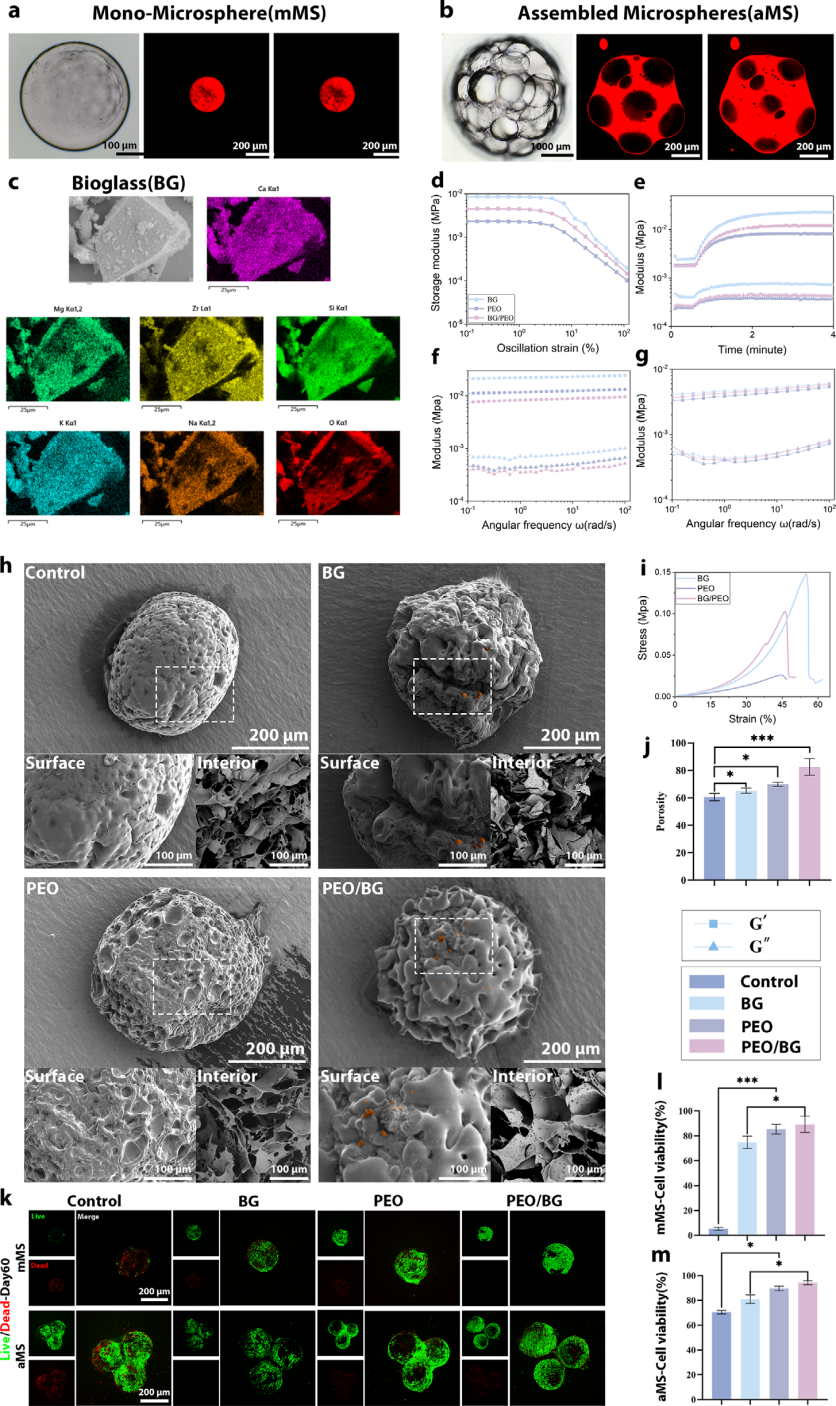
Fabrication, physicochemical characterization, and long‐term cytocompatibility of PEO/BG mono‐microspheres and assembled microspheres. (a) Representative bright‐field and rhodamine fluorescence images of mono‐microspheres (mMS), showing the spherical morphology and homogeneous fluorescent signal. Scale bars, 100 and 200 µm. (b) Representative bright‐field and fluorescence images of assembled microspheres (aMS), showing microsphere clustering and the formation of a larger porous aggregate. Scale bars, 1000 and 200 µm. For fluorescence images in a,b, confocal z‐stacks were acquired at 20 µm intervals and rendered as maximum‐intensity Z‐projections. (c) Scanning electron microscopy image and elemental mapping of bioactive glass (BG), showing the spatial distribution of Ca, Mg, Si, K, Na, and O within the BG particles. Scale bars, 25 µm. (d) Oscillatory strain‐sweep rheology of precursor formulations, showing the storage modulus (G′) and loss modulus (G″) as functions of oscillation strain. (e) Time‐sweep rheology during photocrosslinking, showing rapid gelation and stabilization of G′ and G″ after 405 nm light exposure. (f, g) Frequency‐sweep rheology of microsphere formulations before and after photocrosslinking, showing frequency‐dependent viscoelastic behavior and the predominance of G′ over G″ in crosslinked hydrogel networks. (h) Cryo‐fractured scanning electron microscopy images of freeze‐dried microspheres prepared from four formulations: Control (GelMA), BG (GelMA/BG), PEO (GelMA/PEO), and PEO/BG (GelMA/PEO/BG). Low‐magnification images show overall microsphere morphology, while enlarged views show the outer surface and internal cross‐section. BG particles are indicated by orange pseudocolor. Scale bars, 200 µm and 100 µm. (i) Representative compressive stress–strain curves of microsphere‐derived constructs, demonstrating formulation‐dependent mechanical behavior. (j) Quantification of microsphere porosity across the four formulations, showing increased porosity after PEO incorporation and the highest porosity in the PEO/BG group. (k) Representative live/dead staining images of BMSCs cultured for 60 days in mMS and aMS prepared from the four formulations. Live cells are stained with Calcein‐AM (green), and dead cells are stained with propidium iodide (red). Scale bars, 200 µm. (l, m) Quantification of BMSC viability after 60 days of culture in mMS (l) and aMS (m), showing improved long‐term cytocompatibility in PEO‐ and PEO/BG‐containing microsphere systems. Data are presented as mean ± s.d. (n = 3 independent experiments). Statistical analysis was performed using one‐way ANOVA followed by post hoc multiple‐comparisons testing; *p < 0.05, **p < 0.01, ***p < 0.001.

To verify the inorganic bioactive component incorporated into the microsphere system, we further characterized the morphology and elemental composition of BG particles. SEM imaging showed irregular BG particles with micron‐scale morphology, while elemental mapping demonstrated the homogeneous distribution of Ca, Mg, Si, K, Na, and O throughout the particles (Figure 3c). The presence of Ca, Mg, Si, Na, and K is consistent with the typical ion‐releasing composition of bioactive glass. These results confirmed the successful preparation of multicomponent BG particles and supported their subsequent use as bioactive fillers in the PEO/BG microsphere system.

We next evaluated the rheological behavior of the microsphere precursor formulations and photocrosslinked networks. Oscillatory strain‐sweep measurements showed that the storage modulus (G′) remained higher than the loss modulus (G″) within the linear viscoelastic region, followed by strain‐dependent network disruption at higher oscillation strains (Figure 3d). Time‐sweep rheology during photocrosslinking demonstrated rapid gelation after 405 nm light exposure, as reflected by the sharp increase and subsequent stabilization of G′ and G″, confirming efficient formation of a solid‐like hydrogel network (Figure 3e). Frequency‐sweep analyses further showed that both precursor and cured formulations exhibited formulation‐dependent viscoelastic behavior, while the photocrosslinked hydrogel maintained G′ > G″ across the tested angular‐frequency range, indicating elastic‐dominated network formation after curing (Figure 3f,g). Complementary long‐term physicochemical stability tests showed that all groups underwent rapid early changes from day 1 to day 3, followed by near‐plateau behavior from day 3 to day 14. Compared with the Control and PEO groups, the BG and PEO/BG groups exhibited lower swelling and slower mass loss, suggesting that BG incorporation, particularly in combination with PEO, improved dimensional stability and degradation resistance (Figure S2c,d).

We then investigated how material composition regulated microsphere microarchitecture. Cryo‐fractured SEM images of freeze‐dried microspheres revealed distinct surface and internal structures among the Control (GelMA), BG (GelMA/BG), PEO (GelMA/PEO), and PEO/BG (GelMA/PEO/BG) groups (Figure 3h). Control microspheres displayed a relatively compact morphology, whereas PEO‐containing microspheres exhibited a more open and interconnected porous network, consistent with PEO‐mediated phase separation and leaching. BG incorporation increased surface micro‐roughness, and BG particles were clearly observed within the microsphere matrix, as highlighted by orange pseudocolor (Figure 3h). Quantitative porosity analysis confirmed a composition‐dependent increase in porosity, with the PEO/BG group showing the highest porosity among the four formulations (Figure 3j). These results indicate that PEO and BG play complementary structural roles, with PEO promoting pore formation and microscale interconnectivity, while BG contributes surface roughness and bioactive inorganic cues. The mechanical properties of the microsphere‐derived constructs were further assessed by compression testing and oscillatory deformation. Representative stress–strain curves showed nonlinear compressive responses with clear formulation‐dependent differences (Figure 3i). Compared with PEO alone, the incorporation of BG enhanced the compressive response of the constructs, suggesting that the inorganic phase contributed to mechanical reinforcement. Together with the strain‐ and frequency‐dependent rheological profiles (Figure 3d–g), these data demonstrate that PEO/BG microspheres combine high porosity with sufficient mechanical stability, which is essential for maintaining a permissive 3D scaffold architecture during long‐term culture.

Finally, we assessed whether the structural and mechanical advantages of PEO/BG microspheres translated into improved long‐term cytocompatibility. Live/dead staining after 60 days revealed pronounced formulation‐dependent differences in BMSC survival in both mMS and aMS formats (Figure 3k). Control GelMA mMS showed extensive cell death and poor long‐term viability, whereas BG and PEO incorporation significantly improved BMSC survival (Figure 3k,l). Notably, the PEO/BG mMS group exhibited the highest viability among the mMS formulations, indicating that the combination of porous architecture and BG‐derived bioactivity provided a more favorable microenvironment for sustained cell survival (Figure 3l). Compared with mMS, the aMS format further enhanced long‐term cell viability across formulations, likely owing to its larger interstitial spaces and improved mass transport. PEO/BG aMS achieved the highest BMSC viability among all groups, outperforming the other formulations after 60 days of culture (Figure 3k,m).

These findings provide experimental support for the capacity of the aMS architecture to sustain long‐term cell survival. Compared with discrete mMS, the assembled architecture generated interconnected interstitial spaces throughout the construct, which may improve nutrient and oxygen diffusion, facilitate metabolic waste transport, and reduce local mass‐transport limitations during prolonged culture. The consistently higher BMSC viability observed in the aMS groups, particularly in the PEO/BG aMS group, suggests that microsphere assembly preserves the bioactive advantages of the individual microspheres while providing a more permissive 3D environment for sustained cell maintenance. These results therefore support the use of aMS as a porous and interconnected platform for the subsequent construction of cell‐dense organoid‐like constructs.

Collectively, these results demonstrate that PEO/BG aMS provide a porous, mechanically stable, and cytocompatible 3D niche for long‐term BMSC culture. The combination of multicomponent BG‐derived inorganic bioactivity, PEO‐mediated pore formation, BG‐induced surface roughness, and microsphere assembly‐mediated interstitial architecture supports nutrient diffusion, cell retention, and sustained BMSC survival, establishing PEO/BG aMS as a suitable scaffold platform for subsequent bone–vascular OLCs construction.

### 2.4 PEO/BG aMS Potentiate Bone OLCs Formation and Osteoinductive Signaling

Before constructing bone–vascular **OLCs**, we first examined whether assembled microspheres (aMS) could provide a more favorable 3D microenvironment than mono‐microspheres (mMS) for rapid bone **OLCs** formation and osteoinductive assessment. We refer to these constructs as 3D bone **OLCs** because they combine multicellular aggregation, scaffold‐guided spatial organization, osteogenic marker expression, and matrix mineralization within a porous microstructured niche. Rabbit osteoprogenitors, primary BMSCs, and BMP4‐engineered BMSCs were seeded onto PEO/BG aMS, and osteogenic maturation was evaluated using volumetric confocal z‐stack imaging. Compared with discrete mMS, aMS formed irregular microporous aggregates with extensive interstitial channels, which supported cell infiltration, interconnectivity, and spatial organization within the cluster‐like architecture (Figure 4a,b and Video S1). Accordingly, PEO/BG aMS exhibited a significantly higher cell‐associated BMP2 fluorescence intensity than composition‐matched PEO/BG mMS, indicating increased BMP2 protein expression within the assembled constructs rather than directly demonstrating transcriptional activation (Figure 4c and Videos S2 and S3). The enhanced BMP2 signal observed in aMS may be explained by the convergence of structural, ionic, and multicellular cues. In this system, PEO is considered to contribute primarily through its porogen‐mediated architectural effect rather than through direct osteoinductive activity. The resulting porous microsphere surfaces and interconnected interstitial spaces increase the available cell–material interface and support cell adhesion, spreading, infiltration, and extracellular matrix engagement. Such changes in cell shape and matrix interaction may reinforce cytoskeletal mechanotransduction and BMP/Smad responsiveness, as cell spreading and RhoA/ROCK‐dependent cytoskeletal tension have been shown to regulate BMP‐induced osteogenic signaling in MSCs [27]. Meanwhile, BG dissolution provides bioactive ionic cues that may promote endogenous osteogenic signaling. Previous studies have demonstrated that silica‐based bioactive glasses can increase BMP2 mRNA expression and that BG‐derived ionic products containing Ca‐, Si‐, and Mg‐related species can upregulate BMP2, Runx2, SP7/Osterix, and other osteogenic regulators [28]. Meanwhile, the close spatial organization of cells within aMS may enhance cell–cell communication and strengthen local autocrine and paracrine coupling, consistent with the enhanced osteogenic phenotype reported in 3D MSC aggregates [29]. We therefore propose that the elevated BMP2 protein expression in aMS reflects the combined influence of topology‐mediated cell–matrix mechanosensing, BG‐derived ionic stimulation, and clustering‐dependent multicellular communication.

**FIGURE 4:**
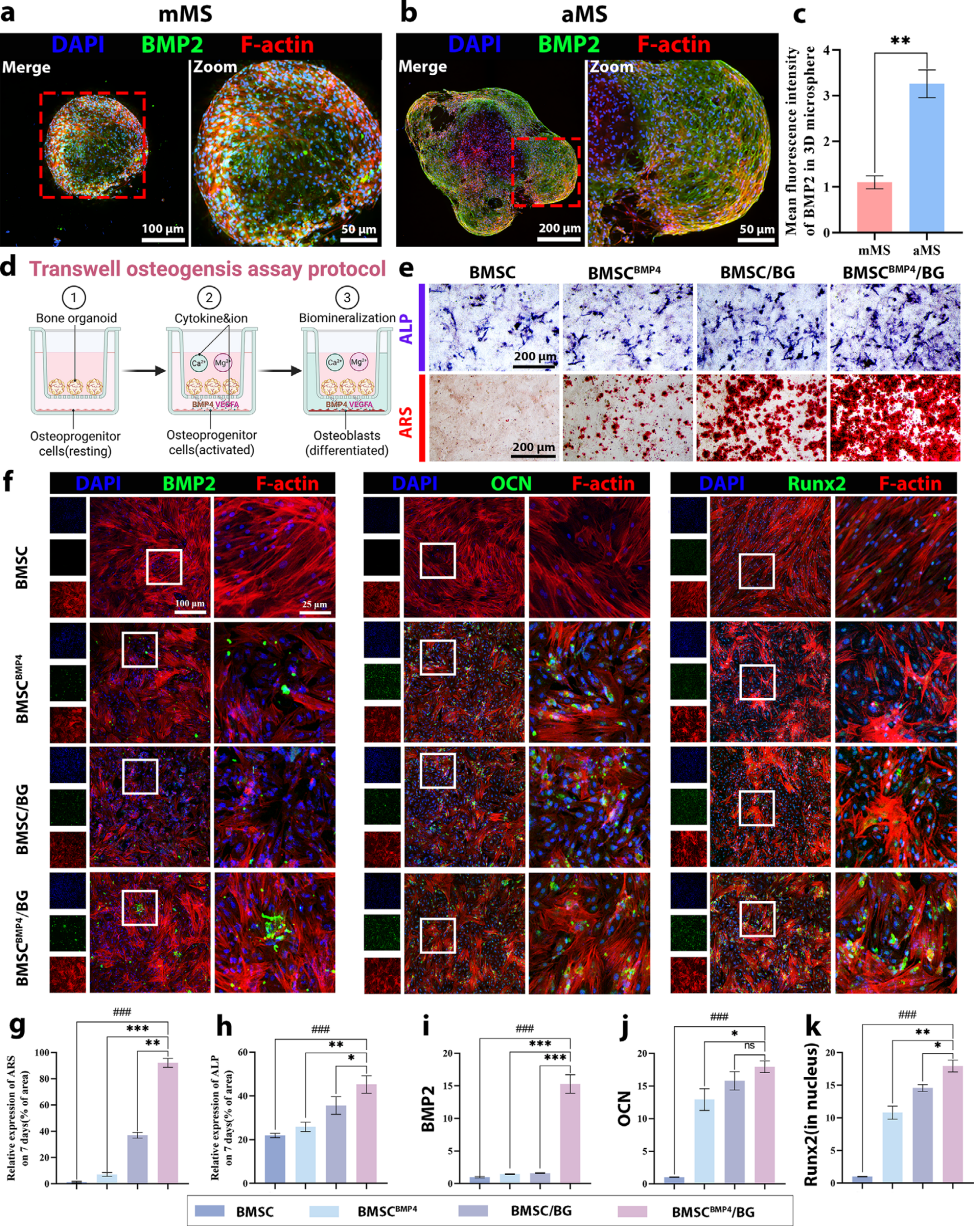
Osteogenic catalytic activity of Bone OLCs. (a) Mono‐microsphere (mMS, Day 5). Confocal Z‐projection after five days of osteogenic culture shows nuclei (DAPI, blue), cytoskeleton (phalloidin, red) and BMP2 (green). Right, enlargement of the dashed region. Scale bars: main = 100 µm; zoom = 50 µm. (b) Assembled‐microsphere cluster (aMS, Day 5). Multiple PEO/BG/GelMA units spontaneously fuse into an irregular macroporous aggregate; identical staining and magnification strategy as in (a). Scale bars: main = 150 µm; zoom = 50 µm. (c) Mean fluorescence intensity (MFI) of BMP2 within mMS versus aMS on Day 5 (n = 3). (d) Schematic of the Transwell assay. (e) Representative Alizarin red S (ARS) and alkaline phosphatase (ALP) staining on day 14; scale bars = 200 µm. (f) Immunofluorescence of BMP2, osteocalcin (OCN) and nuclear Runx2 (green) with F‐actin (phalloidin, red) and nuclei (DAPI, blue); overview scale = 100 µm, inset = 25 µm. (g, h) Densitometric quantification of ARS and ALP (mean ± SD, n = 3). (i‐k) Mean fluorescence intensity for BMP2, OCN and Runx2. Statistical symbols: *p < 0.05, **p < 0.01, ***p < 0.001 vs. BMSC (control); ^##^ *p* < 0.01, ^###^ *p* < 0.001 between experimental groups (one‐way ANOVA).

Tomographic inspection further showed that cells on aMS were more densely distributed and spatially organized than those on mMS, indicating that the interconnected 3D topology provided favorable topographical and spatial cues for multicellular assembly and early osteogenic commitment. These observations further support the biological rationale for constructing aMS rather than using discrete mMS as isolated cell carriers. Specifically, the hierarchical assembly of multiple microspheres increased the overall cell‐supporting surface area and cell‐loading capacity while generating interconnected interstitial spaces throughout the construct. These interstitial channels may facilitate nutrient and oxygen diffusion, promote metabolic waste transport, and alleviate local mass‐transport limitations during prolonged culture. Meanwhile, the close spatial arrangement of neighboring microspheres enabled cells to bridge adjacent surfaces, migrate across microsphere interfaces, and undergo collective aggregation, thereby enhancing cell–cell and cell–matrix interactions. Thus, aMS were designed not merely as aggregates of individual microspheres, but as a porous and interconnected organoid‐supportive niche that integrates efficient mass transport, high cell‐loading capacity, and coordinated multicellular organization, thereby better supporting rapid bone OLCs formation than discrete mMS.

We next used aMS as the primary platform to investigate whether BMP4‐mediated paracrine signaling from BMSC^BMP4^ could synergize with BG‐derived ionic cues to enhance osteoinduction. Supplementary analyses confirmed that, among the BMSC, BMSC^BMP4^, BMSC/BG, and BMSC^BMP4^/BG groups, BMSC^BMP4^/BG **OLCs** showed the highest BMP2 and OCN fluorescence intensities and maintained rapid cell expansion and migration during early culture (Figure S5a–d). These results supported the selection of BMSC^BMP4^/BG as an optimized osteogenic module for subsequent bone **OLCs** construction. To isolate the contribution of soluble paracrine and ionic cues from direct cell–material contact, we established an indirect Transwell co‐culture system (Figure 4d). The upper inserts contained one of four **OLCs** conditions: BMSC, PEO aMS seeded with unmodified BMSCs; BMSC^BMP4^, PEO aMS seeded with BMP4‐engineered BMSCs; BMSC/BG, PEO/BG aMS seeded with unmodified BMSCs; and BMSC^BMP4/BG, PEO/BG aMS seeded with BMP4‐engineered BMSCs.

After 14 days of indirect co‐culture, Alizarin Red S staining revealed abundant mineral deposition in the BMSC^BMP4^/BG group, whereas calcified nodules remained sparse in the BMSC, BMSC^BMP4^, and BMSC/BG groups (Figure 4e). Quantification showed that the ARS‐positive area in BMSC^BMP4^/BG increased by approximately ninefold relative to BMSC^BMP4^ and by approximately threefold relative to BMSC/BG, indicating that BMP4‐driven osteogenic priming requires a mineral‐supportive ionic microenvironment to efficiently progress toward matrix mineralization (Figure 4g). Consistently, ALP staining and area quantification showed the highest enzymatic activity in BMSC^BMP4^/BG, with 2.1‐fold and 1.7‐fold increases over BMSC^BMP4^ and BMSC/BG, respectively (Figure 4e,h). These results indicate that BMP4 paracrine stimulation and BG‐derived ionic cues act cooperatively to enhance early osteogenic commitment and mineralization.

To further validate osteogenic differentiation, we performed immunofluorescence staining for BMP2, Runx2, and OCN, representing early‐to‐mid osteogenic signaling, early transcriptional regulation, and late osteogenic maturation, respectively (Figure 4f). The BMSC group exhibited weak BMP2, Runx2, and OCN signals, whereas all three markers were most strongly expressed in BMSC^BMP4^/BG **OLCs**. Quantitative analysis confirmed that the mean fluorescence intensities of BMP2, OCN, and Runx2 were significantly higher in BMSC^BMP4^/BG than in the other groups (Figure 4i–k). Notably, BMP2 upregulation in BMSC/BG was relatively modest, supporting the key contribution of BMP4‐mediated paracrine signaling to early osteogenic activation. In contrast, the increases in OCN and Runx2 in BMSC/BG suggest that BG‐derived ionic cues may contribute to osteogenic maturation and matrix development.

Together, these results demonstrate that PEO/BG aMS‐based bone **OLCs** integrate BMP4‐mediated paracrine priming with BG‐derived ionic stimulation to accelerate osteogenic commitment, matrix maturation, and mineral deposition. This biomaterials and cell synergistic design establishes BMSC^BMP4^/BG‐loaded PEO/BG aMS as an optimized osteoinductive module for subsequent bone–vascular **OLCs** construction.

### 2.5 Integrated Transcriptomic and Protein‐Level Analyses Reveal Cooperative BMP4/BG‐Driven Osteogenic Activation

To define how BMP4‐engineered BMSCs and BG‐containing assembled microspheres jointly modulate osteogenic maturation, we performed bulk transcriptomic profiling of four microsphere constructs: BMSC, BMSC^BMP4^, BMSC/BG, and BMSC^BMP4^/BG. Principal component analysis showed clear segregation among the four groups along PC1 and PC2, which together explained 97.9% of the total variance. Notably, the BMSC^BMP4^/BG group was positioned far from the BMSC control and was also distinct from the single‐factor BMSC^BMP4^ and BMSC/BG groups, indicating that combined BMP4 and BG stimulation induced the most pronounced and directionally distinct transcriptional shift within the osteogenic program (Figure S7a). Consistently, immunofluorescence staining of day‐5 bone OLCs showed markedly enhanced BMP2 and OCN signals in BMSC ^BMP4^/BG compared with BMSC, accompanied by dense cytoskeletal organization within the OLCs structure (Figure 5a,b). A broader comparison across all experimental groups further confirmed this graded enhancement of osteogenic marker expression, with the BMSC^BMP4^/BG group exhibiting the strongest BMP2 and OCN signals among the tested constructs (Figure S5a, b).

**FIGURE 5:**
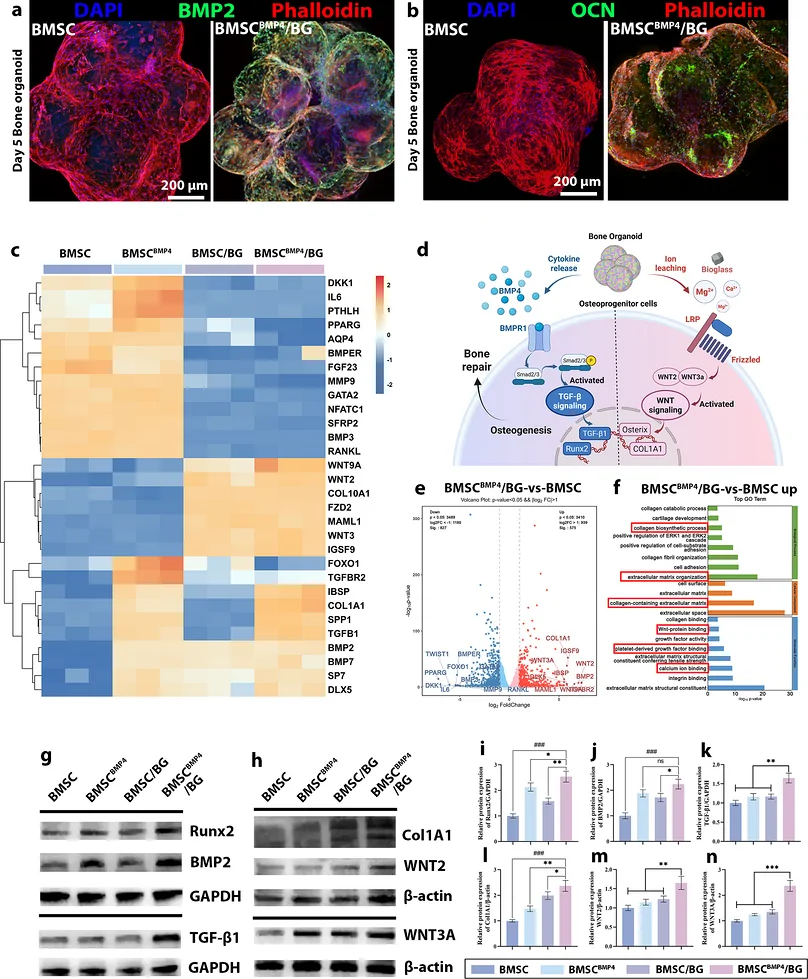
Integrated transcriptomic and protein‐level analyses identify candidate signaling programs associated with osteogenic maturation driven by BMSC^BMP4^‐loaded PEO/BG aMS. (a, b) Immunofluorescence staining of day‐5 bone OLCs showing enhanced BMP2 (a) and OCN (b) expression in the BMSC^BMP4^/BG group compared with the BMSC group. Nuclei are stained with DAPI, and cytoskeletal organization is visualized by phalloidin staining. (c) Heatmap of osteogenesis‐related gene expression across four groups: BMSC, BMSC^BMP4^, BMSC/BG, and BMSC^BMP4^/BG. The BMSC^BMP4^/BG group shows coordinated upregulation of osteogenic, extracellular matrix, and signaling‐associated genes, including *BMP2*, *SPP1*, *IBSP*, *COL1A1*, *RUNX2*, and Wnt‐related genes. (d) Hypothesized working model by which BMSC^BMP4^/BG OLCs may promote bone repair. BMP4 released from engineered BMSCs may activate BMPR‐mediated Smad signaling, whereas bioactive glass‐derived ions, including Mg^2^^+^ and Ca^2^^+^, may contribute to osteogenic stimulation and Wnt pathway activation. These candidate signaling events may collectively enhance osteogenic differentiation and extracellular matrix deposition. (e) Volcano plot of differentially expressed genes between BMSC^BMP4^/BG and BMSC groups. Upregulated genes are shown in red and downregulated genes in blue; representative osteogenesis‐related genes, including *COL1A1*, *IGSF9*, *WNT2*, *WNT3A*, *BMP2*, *SPP1*, and *RUNX2*, are highlighted. (f) Gene Ontology enrichment analysis of upregulated differentially expressed genes in the BMSC^BMP4^/BG group, showing enrichment of extracellular matrix organization, collagen‐containing extracellular matrix, collagen fibril organization, cell adhesion, growth factor binding, and Wnt‐related biological processes. (g, h) Western blot validation of osteogenesis‐related proteins. Runx2, BMP2, and TGF‐β1 are shown in (g), while Col1A1, WNT2, and WNT3A are shown in (h). GAPDH or β‐actin was used as the loading control. Lane order: BMSC, BMSC^BMP4^, BMSC/BG, and BMSC^BMP4^/BG. (i–n) Quantitative analysis of Runx2 (i), BMP2 (j), TGF‐β1 (k), Col1A1 (l), WNT2 (m), and WNT3A (n) protein expression levels, normalized to GAPDH or β‐actin. Data are presented as mean ± SD, n = 3 independent samples. Statistical significance was determined by one‐way ANOVA with post hoc multiple‐comparison analysis. #*p* < 0.05, ##*p* < 0.01, and ###*p* < 0.001 versus the BMSC control group; *p < 0.05, **p < 0.01, and ***p < 0.001 for the indicated comparisons; ns, not significant.

We next examined whether this global transcriptional separation was associated with coordinated regulation of osteogenesis‐related genes. The heatmap showed that BMSC^BMP4^/BG constructs exhibited broad upregulation of osteogenic differentiation, extracellular matrix, and signaling‐associated genes, including *BMP2, SPP1, IBSP, COL1A1, RUNX2, WNT2, WNT3A*, and other Wnt and matrix‐related genes (Figure 5c). Compared with either BMP4 engineering or BG stimulation alone, the combined BMSC^BMP4^/BG group displayed a more coherent osteogenic gene‐expression pattern, suggesting that the two cues cooperatively reinforced the transcriptional program required for bone matrix development and maturation.

Differential expression analysis further supported this cooperative transcriptional remodeling. Using |log_2_FC| > 1 and FDR < 0.05 as the threshold, the volcano plot comparing BMSC^BMP4^/BG with BMSC revealed a distinct set of upregulated genes associated with osteogenesis and extracellular matrix formation, including *COL1A1, IGSF9, WNT2, WNT3A, BMP2, SPP1*, and RUNX2 (Figure 5e). Functional enrichment analysis of the upregulated differentially expressed genes showed significant enrichment of extracellular matrix organization, collagen‐containing extracellular matrix, collagen fibril organization, cell adhesion, growth factor binding, and Wnt‐related biological processes (Figure 5f). KEGG enrichment analysis further indicated enrichment of ECM–receptor interaction, PI3K–Akt, TGF‐β, and Wnt‐related pathways, while pathway–gene mapping suggested extensive connections among ECM remodeling, growth factor signaling, and Wnt‐associated nodes (Figure S7b, c). These results support a model in which BMP4/BG co‐stimulation enhances osteogenic maturation through coordinated engagement of matrix‐associated signaling, growth‐factor pathways, and Wnt‐related transcriptional programs.

To validate these transcriptomic findings at the protein level, we performed Western blot analysis of representative markers involved in osteogenic transcriptional regulation, osteoinductive cytokine signaling, matrix deposition, and Wnt‐associated signaling. Runx2, BMP2, and TGF‐β1 showed the strongest protein bands in the BMSC^BMP4^/BG group, with intermediate expression in the BMSC^BMP4^ and BMSC/BG groups and the weakest expression in the BMSC control (Figure 5g). Similarly, Col1A1, WNT2, and WNT3A were most prominently expressed in BMSC^BMP4^/BG constructs, supporting enhanced extracellular matrix assembly and Wnt‐associated osteogenic signaling (Figure 5h). Quantitative analysis confirmed that Runx2, BMP2, TGF‐β1, Col1A1, WNT2, and WNT3A were elevated in BMSC^BMP4^/BG compared with BMSC, with several markers also significantly higher than those in the single‐factor BMSC^BMP4^ or BMSC/BG groups (Figure 5i–n). These protein‐level results were consistent with the transcriptomic enrichment data, indicating concurrent reinforcement of osteogenic transcription, BMP/TGF‐β‐related signaling, collagen matrix formation, and Wnt‐associated pathways.

On the basis of these findings, we propose a hypothetical working model for BMSC^BMP4^/BG‐mediated maturation of bone OLCs (Figure 5d). BMP4 secreted by engineered BMSCs may activate BMPR‐mediated Smad signaling and thereby support Runx2/Osterix‐associated osteogenic transcription. In parallel, ionic cues released from BG, including Ca^2^^+^ and Mg^2^^+^, together with adhesion‐dependent extracellular matrix sensing, may contribute to the regulation of Wnt‐associated and PI3K–Akt‐related signaling through matrix‐interaction and LRP/Frizzled‐associated pathways. This candidate signaling events may collectively favor osteogenic gene expression, collagen I deposition, extracellular matrix organization, and mineral‐associated maturation. Accordingly, self‐assembling PEO/BG aMS loaded with BMP4‐engineered BMSCs may provide a cooperative osteoinductive microenvironment in which osteogenic priming and bioactive ionic stimulation potentially reinforce ECM–receptor interaction‐, PI3K–Akt‐, TGF‐β‐, and Wnt‐associated signaling. This proposed model is supported by transcriptomic enrichment, gene‐expression analysis, and protein‐level validation, but does not establish direct causal relationships among these pathways. Further studies using pathway‐specific inhibitors, receptor blockade, or targeted gene knockdown will be required to determine their causal contributions. Nevertheless, the present findings provide a rationale for selecting BMSC^BMP4^/BG‐loaded PEO/BG aMS as the osteogenic core for subsequent bone–vascular **OLCs** assembly and in vivo bone‐defect repair.

### 2.6 BMSC^BMP4+VEGFA^ Paracrine Cues Induce a Pro‐Angiogenic VEC Phenotype

To determine whether paracrine factors released by BMP4+VEGFA–engineered BMSCs (BMSC^BMP4+VEGFA^) can drive vascular endothelial cells (VECs) toward a pro‐angiogenic state without direct cell–cell contact, we established an indirect co‐culture/conditioned‐medium (CM) system (Figure 6a). Following a predefined mechanistic sequence, we assessed VEC behavior, including migration, proliferation, angiogenic morphogenesis in 2D and 3D matrices, and phenotypic switching at the population level. Transwell migration assays were performed to evaluate VEC motility in response to graded concentrations of BMSC^BMP4+VEGFA^‐conditioned medium. VECs were seeded in the upper chambers, whereas the lower chambers contained basal medium or 10%, 25%, or 50% conditioned medium. After 24 and 48 h, cells that had migrated to the lower surface of the membrane were stained with crystal violet and quantified. BMSC^BMP4+VEGFA^‐conditioned medium increased VEC migration in a concentration‐ and time‐dependent manner, with the most pronounced response observed after 48 h (Figure S8). These findings indicate that factors derived from BMSC^BMP4+VEGFA^ effectively enhance VECs migration, a prerequisite for tip‐cell sprouting.

**FIGURE 6:**
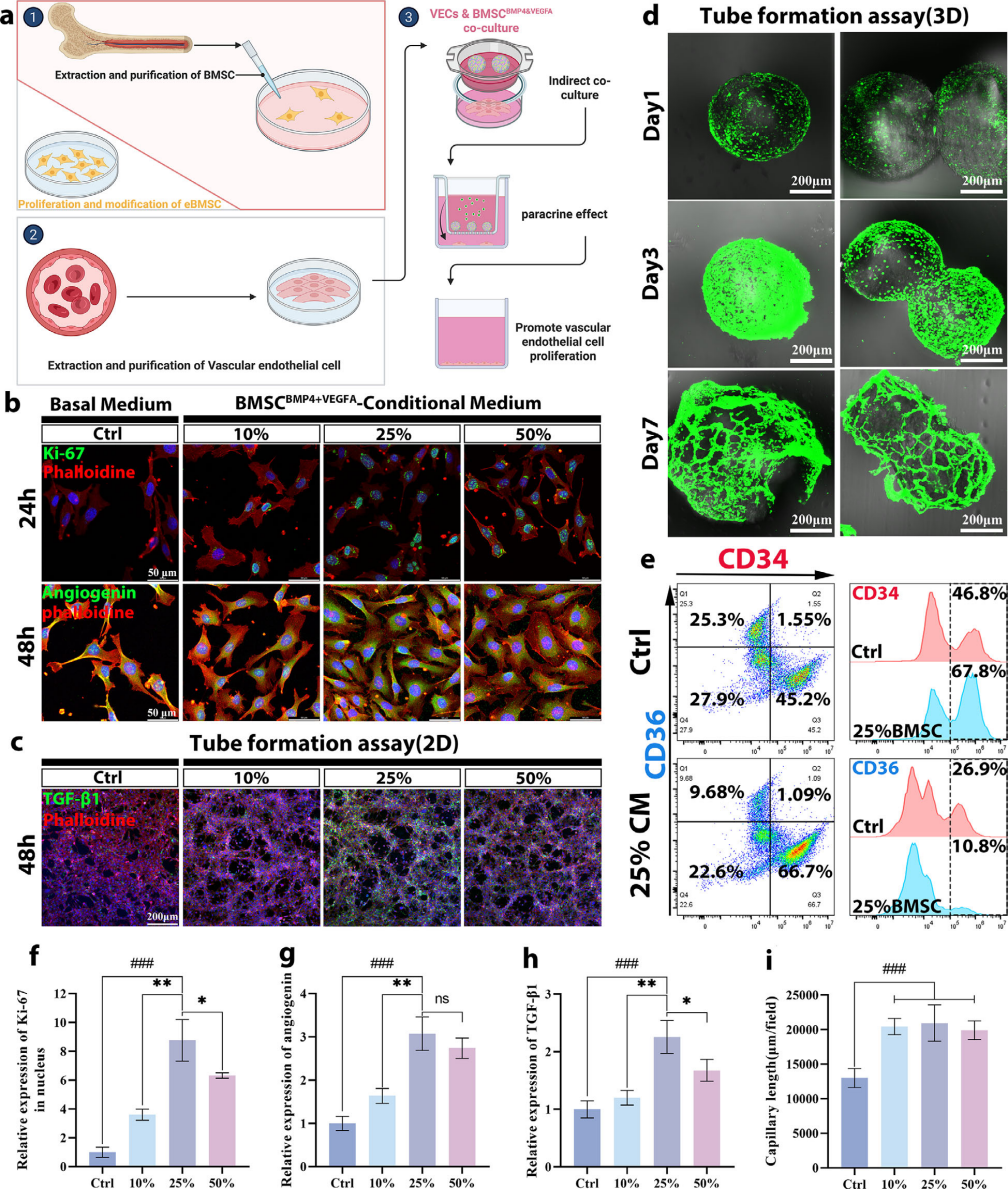
BMSC^BMP4+VEGFA^‐derived paracrine cues enhance proliferation and angiogenic differentiation of vascular endothelial cells (VECs). (a) Experimental design. Bone‐marrow mesenchymal stem cells (BMSCs) were isolated and transiently transfected with BMP4‐ and VEGFA‐encoding plasmids to generate BMSC^BMP4+VEGFA^, primary VECs were isolated, and paracrine effects were assessed using indirect Transwell co‐culture or treatment with conditioned medium (CM). (b) Immunofluorescence of VECs treated with the indicated CM concentrations. Ki‐67 (green) and phalloidin (red) at 24 h (top), and angiogenin (green) and phalloidin (red) at 48 h (bottom). Scale bar, 50 µm. (c) 2D Matrigel tube‐formation assay at 48 h. VECs were stained for TGF‐β1 (green) and phalloidin (red). Scale bar, 200 µm. (d) 3D sprouting assay. GFP‐labeled VEC spheroids embedded in fibrin were imaged by confocal microscopy; maximum‐intensity projections at days 1, 3 and 7 show progressive microvascular branching. Scale bar, 200 µm. (e) Flow‐cytometric analysis of CD34 and CD36 expression in VECs treated with 25% BMSC^BMP4+VEGFA^‐CM for 72 h compared with control. (f–h) qRT–PCR analysis of *Ki‐67* (f), *angiogenin* (g) and *TGF‐β1* (h) mRNA levels in VECs after 48 h CM treatment (n = 3 independent experiments). (i) Quantification of total capillary length in the 2D tube‐formation assay (n = 5 independent experiments). Data are mean ± s.d. Statistical analysis was performed using one‐way ANOVA with Tukey's multiple‐comparisons test. Significance is indicated as follows: ^###^ *p* < 0.001, ^##^ *p* < 0.01 and ^#^ *p* < 0.05 versus control; ** *p* < 0.01 and * *p* < 0.05 between the indicated experimental groups; ns, not significant.

We next examined whether increased motility is accompanied by cell‐cycle entry and upregulation of pro‐angiogenic signals. After 48 h of CM exposure, immunofluorescence showed a higher fraction of Ki‐67–positive cells together with stress‐fiber remodeling and increased cell spreading (Figure 6b,f), consistent with increased cell proliferation. In parallel, angiogenin staining intensity was increased at 48 h, indicating enhanced pro‐angiogenic signaling (Figure 6b,g). To further determine whether these changes translate into functional tubulogenesis, we plated VECs on Matrigel and co‐seeded them in the same wells with different proportions of BMSC^BMP4+VEGFA^. After 48 h, spontaneous tube formation was observed under all conditions, but the 25% BMSC^BMP4+VEGFA^ group exhibited the greatest sprouting vessel length, indicating the strongest tubulogenic activity (Figure 6c,i). Consistent with the enhanced angiogenic phenotype, TGF‐β1 expression in this group was higher than that in the other groups (Figure 6c,h). Taken together, these results indicate that a 25% fraction of BMSC^BMP4+VEGFA^ most effectively promotes VEC proliferation while providing the strongest induction of angiogenic tube formation.

To evaluate tissue‐relevant architecture, we performed GFP‐labeled VECs spheroid assays in 3D fibrin gels(Figure 6d). By Day 1, radial primary sprouts emerged; by Day 3, secondary branches appeared and elongated; by Day 7, a hierarchical, interconnected microvascular network had formed. These 3D results demonstrate that BMSC^BMP4+VEGFA^‐CM sustains branching morphogenesis and network remodeling within a physiologically relevant matrix.

Finally, to assess a population‐level shift from quiescent to activated endothelium, flow cytometry after 72 h in 25% CM showed a significant increase in CD34^high^ cells with a concomitant decrease in CD36 expression relative to Ctrl (Figure 6e). This shift from a CD34^low^/CD36^high^ resting profile to a CD34^high^/CD36^low^ activated phenotype indicates that paracrine cues can reprogram endothelial state to favor angiogenesis. Across multiple experimental readouts, BMSC^BMP4+VEGFA^ paracrine signaling significantly enhances VEC migration and proliferation, upregulates angiogenin and TGF‐β1 pathways, promotes robust tube formation in 2D and sustained sprouting and network assembly in 3D, and converts endothelial cells to an activated CD34^high^/CD36^low^ phenotype. These findings support the mechanistic model: engineered microspheres provide sustained VEGFA/BMP4 release that converts quiescent VECs into sprouting‐competent activated cells, thereby accelerating neovascularization and establishing a strong foundation for rapid, stable vascularization in OLCs and in vivo applications.

### 2.7 BMSC^BMP4+VEGFA^ Promote Vascular‐Like Endothelial Sprouting in Bone–Vascular OLCs

To determine whether BMP4/VEGFA‐engineered BMSCs could promote endothelial sprouting within composite bone–vascular OLCs, we co‐assembled BMSCs or BMSC^BMP4+VEGFA^ with vascular endothelial cells (VECs) and osteoprogenitor cells (OPCs) on microsphere clusters in low‐adhesion U‐bottom wells. The multicellular aggregates were subsequently cultured in DMEM/F12 containing 2% GelMA for 72 h to provide a low‐density supportive matrix for paracrine signaling, cell migration, and vascular‐like morphogenesis (Figure 7a).

**FIGURE 7:**
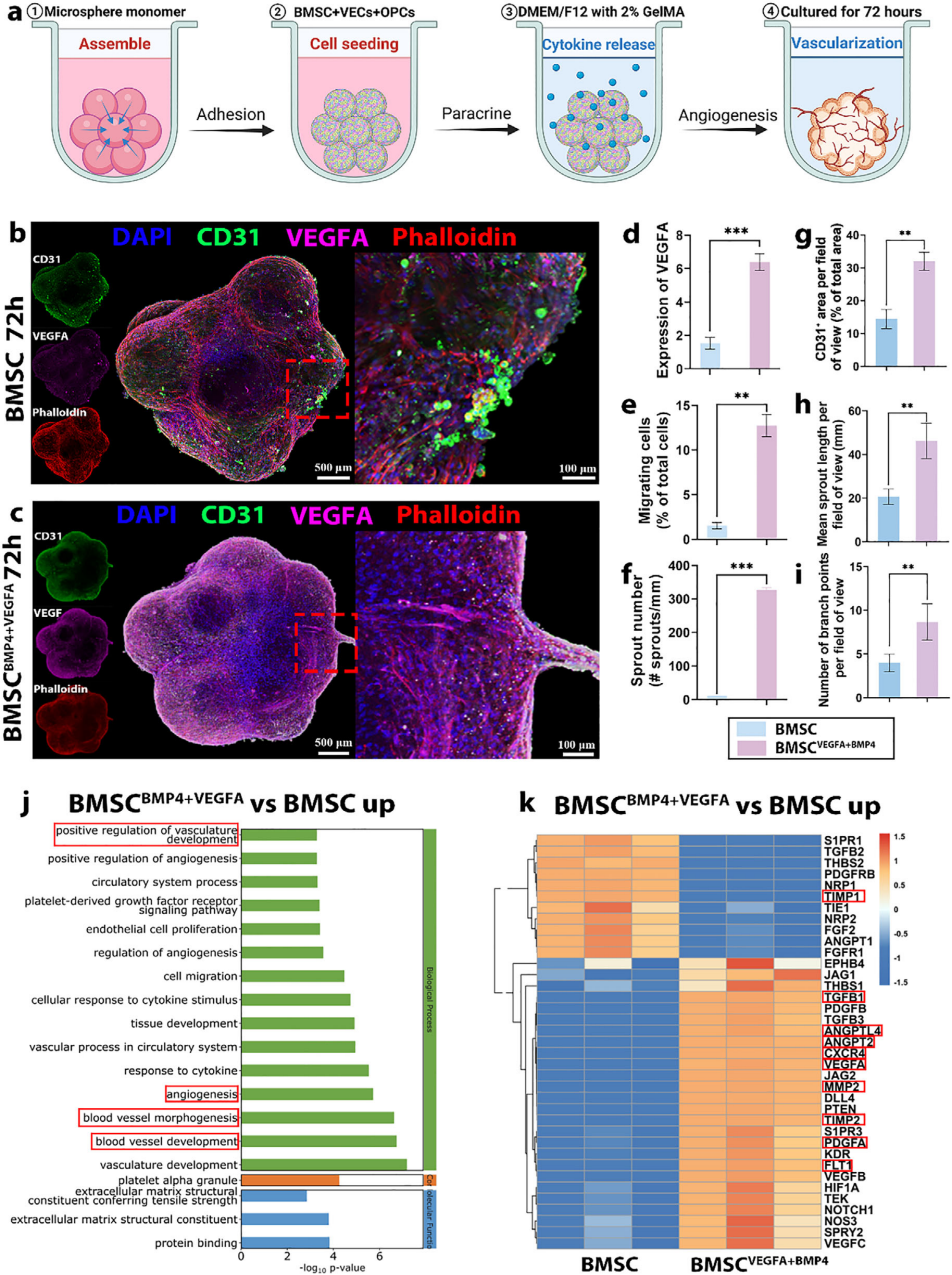
Vascular‐like sprouting and angiogenic transcriptomic activation in BMSC‐derived OLCs. (a) Schematic illustration of the bone–vascular OLCs construction workflow. Microsphere monomers were first assembled into clusters in low‐adhesion U‐bottom plates, followed by co‐seeding with BMSCs or BMSC^BMP4+VEGFA^, vascular endothelial cells (VECs), and osteoprogenitor cells (OPCs). The cell‐laden microsphere clusters were then embedded/cultured in DMEM/F12 containing 2% GelMA for 72 h, during which cytokine release and paracrine interactions promoted vascular‐like network formation. (b, c) Whole‐mount confocal images of OLCs after 72 h of culture in the BMSC control group (b) and BMSC^BMP4+VEGFA^ group (c). Samples were stained for CD31, VEGFA, phalloidin, and DAPI to visualize endothelial cells, angiogenic factor expression, cytoskeletal organization, and nuclei, respectively. Left insets show separated fluorescence channels, and right panels show magnified views of the boxed regions. Scale bars, 500 µm in overview images and 100 µm in magnified images. (d–i) Quantitative analysis of vascular‐like organization in BMSC and BMSC^BMP4+VEGFA^ OLCs, including relative VEGFA expression (d), percentage of migrating cells (e), sprout number (f), CD31‐positive area per field of view (g), mean sprout length per field of view (h), and number of branch points per field of view (i). (j) Gene Ontology enrichment analysis of upregulated transcripts in BMSC^BMP4+VEGFA^ OLCs compared with BMSC OLCs, showing enrichment of angiogenesis‐associated processes, including positive regulation of vasculature development, endothelial cell proliferation, regulation of angiogenesis, cell migration, blood vessel morphogenesis, blood vessel development, and vasculature development, as well as extracellular matrix‐related molecular functions. Bars represent −log10 *p* values. (k) Heatmap of selected differentially expressed genes associated with angiogenesis, endothelial activation, growth factor signaling, and extracellular matrix remodeling in BMSC and BMSC^BMP4+VEGFA^ OLCs. Upregulated genes in the BMSC^BMP4+VEGFA^ group include angiogenesis‐related factors such as *VEGFA, ANGPT1/2, PDGFB, PDGFA, HGF, ITGB1, ITGB5, TEK, MMP2, MMP3, SPP1, and ITGA3*. Color scale indicates relative gene expression. Data are presented as mean ± SD, *n* = 3 independent samples. Statistical significance was determined by unpaired two‐tailed Student's *t*‐test or one‐way ANOVA where appropriate. *P < 0.05, **p < 0.01 and ***p < 0.001.

Whole‐mount confocal imaging revealed clear differences in endothelial organization between the two groups. In BMSC control OLCs, CD31^+^ endothelial cells were sparsely distributed and showed limited outward migration from the OLCs body (Figure 7b). In contrast, BMSC^BMP4+VEGFA^ OLCs exhibited markedly stronger VEGFA signals, more extensive CD31^+^ endothelial cell distribution, and prominent vascular‐like protrusions extending from the OLCs surface (Figure 7c, Video S4, “Bone–Vascular OLCs‐72 h”). Magnified views further showed that CD31^+^ cells accumulated along the peripheral region and formed sprout‐like extensions within the GelMA‐supported microenvironment, indicating that BMP4/VEGFA engineering promoted endothelial mobilization and early vascular‐like patterning.

Quantitative analysis confirmed these imaging observations. VEGFA fluorescence intensity was significantly higher in BMSC^BMP4+VEGFA^ OLCs than in BMSC controls, demonstrating enhanced local angiogenic‐factor expression within the engineered constructs (Figure 7d, Figure S15). The proportion of migrating cells was also significantly increased in the BMSC^BMP4+VEGFA^ group, suggesting that the engineered paracrine cues promoted cellular redistribution and outward migration into the surrounding matrix (Figure 7e). Consistently, the number of endothelial sprouts was markedly elevated following dual BMP4/VEGFA engineering (Figure 7f). Further morphometric analysis revealed a significantly greater CD31‐positive area per field of view, increased mean sprout length, and a higher number of branch points in BMSC^BMP4+VEGFA^ OLCs than in BMSC controls (Figure 7g–i). Collectively, these results indicate that dual BMP4/VEGFA engineering enhances not only angiogenic‐factor expression and cell migration, but also the extent, elongation, and branching complexity of vascular‐like structures within the developmental microenvironment.

To further link the observed phenotype with molecular regulation, we compared the transcriptomic profiles of BMSC and BMSC^BMP4+VEGFA^ OLCs. GO enrichment analysis of upregulated transcripts in the BMSC^BMP4+VEGFA^ group showed significant enrichment of angiogenesis‐associated terms, including positive regulation of vasculature development, regulation of angiogenesis, endothelial cell proliferation, cell migration, blood vessel morphogenesis, blood vessel development, and vasculature development (Figure 7j). In addition, extracellular matrix‐related terms, including extracellular matrix structural constituent and protein binding, were enriched, suggesting that endothelial sprouting was accompanied by matrix remodeling and cell–matrix interaction.

A focused heatmap further demonstrated coordinated upregulation of genes involved in angiogenic growth‐factor signaling, endothelial activation, vascular remodeling, and extracellular matrix organization (Figure 7k). These included VEGF‐associated genes and receptors or co‐receptors, such as *VEGFA, KDR, NRP1, and NRP2*; vascular stabilization and maturation‐related genes, including *ANGPT1, ANGPT2, TEK, PDGFA, PDGFB, and PDGFRB*; Notch‐associated regulators, including *NOTCH1, DLL4, JAG1, and JAG2*; and matrix‐remodeling or adhesion‐related genes, including *MMP2, TIMP1, TIMP2, ITGB1, ITGB5, ICAM1, THBS1, THBS2, SPP1*, and *S1PR1*. This coordinated transcriptional pattern supports a mechanism in which BMSC^BMP4+VEGFA^‐derived cues promote endothelial activation through integrated VEGF‐related signaling, vessel maturation pathways, Notch‐associated regulation, and extracellular matrix remodeling.

Together, these structural, quantitative, and transcriptomic results demonstrate that BMSC^BMP4+VEGFA^ OLCs establish a pro‐angiogenic microenvironment within 72 h, characterized by enhanced VEGFA expression, increased endothelial migration, elevated sprout formation, and activation of a coherent vascular morphogenesis gene program. These findings support the use of BMSC^BMP4+VEGFA^‐containing OLCs as a vascular‐inductive module for constructing bone–vascular OLCs with self‐organizing vascular‐like networks.

### 2.8 Generation of a Mature Femoral Organoid‐Like Constructs With 3D Vascular‐Like Microchannels

To generate and validate a mature femoral OLCs with 3D, vascular‐like microchannels, we applied a stepwise evaluation of microsphere architecture, multicellular cooperation, paracrine signaling programs, and tissue remodeling (Figure 8a). Our first objective was to determine whether BMSC^BMP4+VEGFA^ drives early capillary‐like morphogenesis. Vascular endothelial cells (VECs), osteoprogenitors, and BMSC^BMP4+VEGFA^ were co‐aggregated in low‐adhesion U‐bottom wells and cultured in DMEM/F12 supplemented with 2% GelMA. Whole‐mount confocal imaging was performed on days 3, 7, and 14 according to the experimental workflow. As shown in Figure 8b,c, compared with control BMSC aggregates, BMSC^BMP4+VEGFA^ formed a well‐organized 3D vascular‐like network accompanied with elevated VEGFA and ANGPT2 expression.

**FIGURE 8:**
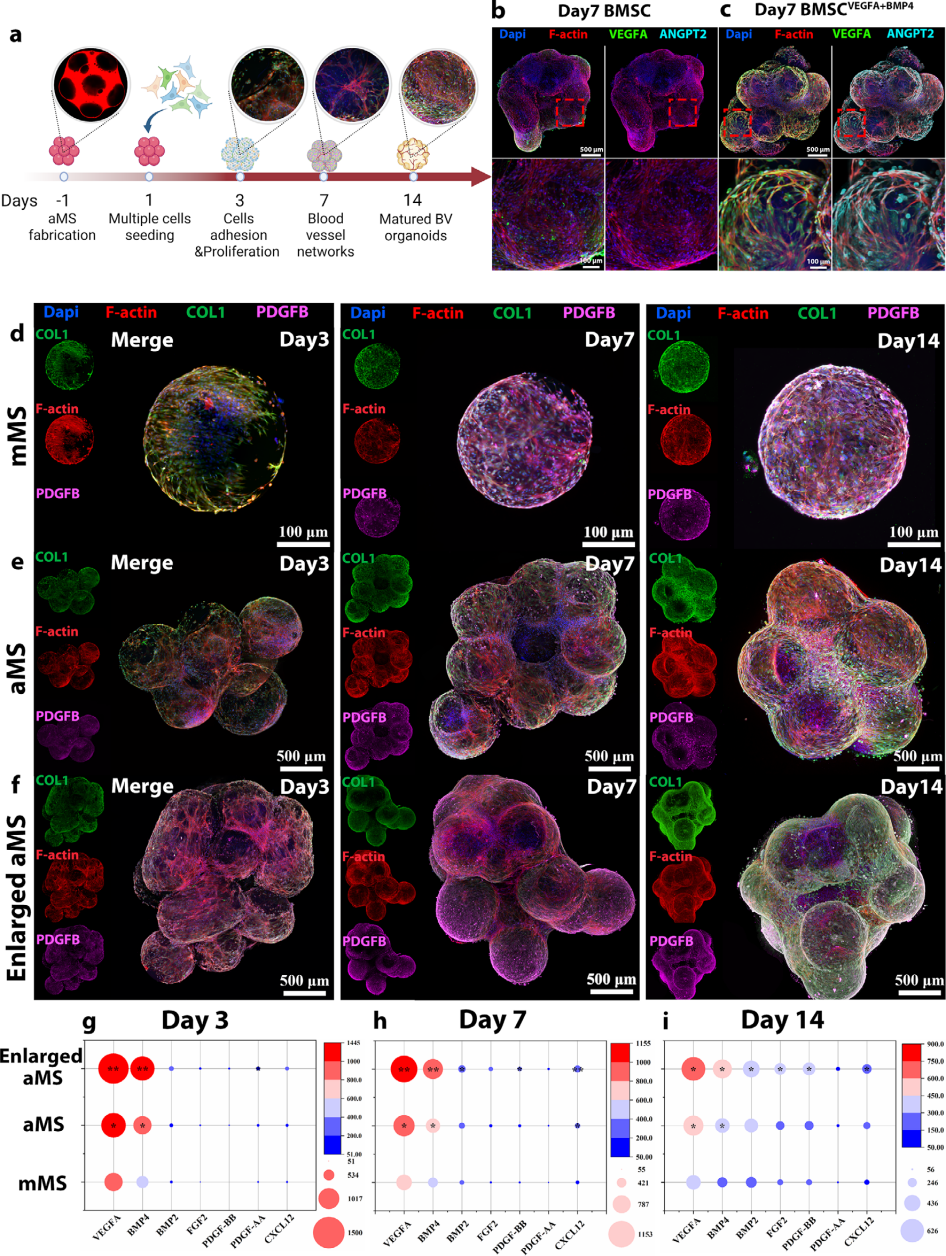
Generation of a mature bone–vascular OLCs with 3D vascular‐like microchannels. (a) Schematic workflow of femoral OLCs formation and maturation. The mMS were produced (Day ‐1) and subsequently seeded (Day 1) with VECs, osteoprogenitor cells and BMSC^BMP4+VEGFA^ co‐overexpressing **VEGFA** and **BMP4**. OLCs underwent (Day 3) cell adhesion/proliferation, (Day 7) capillary‐like network formation, and (Day 14) maturation into a 3D vascular‐like architecture. (b, c) Confocal images of Day7 OLCs seeded with control BMSC or BMSC^BMP4+VEGFA^, illustrating nascent capillary structures and the colocalized expression of vascular proteins **VEGFA** and **ANGPT2**. Staining: DAPI (nuclei, blue), VEGFA (green), phalloidin (F‐actin, red) and ANGPT2 (cyan). Scale bars: 500 µm in overview images, 100 µm in magnified insets. (d–f) Time‐lapse morphology and matrix remodeling of OLCs fabricated from mMS, aMS and enlarged aMS at Days 3, 7 and 14. Immunofluorescence shows co‐expression of type I collagen (**COL I**, green) and **PDGFB** (magenta) with F‐actin (red). Nuclei are counter‐stained with DAPI (blue). Scale bars: 500 µm in aMS and Enlarged aMS images, 100 µm in mMS images. (g–i) Bubble plots of cytokine secretion (VEGFA, BMP4, BMP2, FGF2, PDGFBB, PDGFAA and CXCL12) from OLCs constructed with mMS, aMS and enlarged aMS on Days 3, 7 and 14, respectively (**p < 0.01; *p < 0.05; one‐way ANOVA with Tukey post‐hoc test).

To determine how microsphere construction influences histological maturation and matrix remodeling, we compared OLCs assembled from mono‐microsphere (mMS), assembled‐microsphere (aMS), and enlarged aMS at days 3/7/14. By day 3, all conditions showed peripheral type I collagen (COL I) deposition and F‐actin tension fibers; however, aMS and enlarged aMS had already formed interconnected macroporous channels. By day 7, both aMS and enlarged aMS displayed COL I banding and aligned cellular bundles tracking along the channels. By day 14, COL I in the aMS systems condensed into continuous sheath‐like structures that enveloped PDGFB^+^ cell clusters (Figure 8e, Day14 Middle, Video S5), whereas mMS remained composed of discrete micro‐islands (Figure 8d–f). Co‐expression of PDGFB and COL I indicates synchronized matrix deposition with pericyte recruitment/stabilization, consistent with the temporal progression of vascular‐like microchannels formation. The presence of macropores and interconnecting conduits explains the improved nutrient and signaling diffusion in self‐assembling constructs, which accelerates tissue‐level anisotropic remodeling.

To examine the relationship between engineered cells, microenvironmental cues, and OLCs morphology, we longitudinally profiled secretomes across architectures. Cytokines quantification in conditioned media showed that VEGFA, BMP4, and BMP2 were elevated as early as day 3, with higher levels in aMS and enlarged aMS than in mMS, and the greatest increases observed in enlarged aMS (Figure 8g). By day 7, FGF2 and PDGF‐BB/PDGF‐AA further increased, with sustained or amplified advantages in the aMS and enlarged aMS (Figure 8h), consistent with the expanded vascular‐like networks observed at this time point. By day 14, most pro‐angiogenic and pro‐osteogenic factors remained at high levels in the aMS and enlarged aMS systems, whereas mMS showed more pronounced decline (Figure 8i). Persistent CXCL12 secretion provides a chemotactic basis for directed endothelial/pericyte recruitment and lumen stabilization, suggesting that architectural scale and connectivity influence both paracrine flux and local retention to amplify angiogenic and osteogenic signaling.

Collectively, these data suggest that BMSC^BMP4+VEGFA^ promote early endothelial sprouting through VEGF and ANG/Tie signaling. In parallel, aMS architectures, particularly larger clusters, leverage a macropore‐interconnected topology to accelerate COL1–PDGFB–driven matrix remodeling and canal stabilization, while sustaining high‐level secretion of VEGFA, BMP4, BMP2, FGF2, PDGF, and CXCL12. Evidence from transcriptomics, secretome profiling, and histology supports a unified mechanism: coupling dual‐gene‐engineered cells with an embryo‐mimetic self‐assembling scaffold converts mixed cell aggregates into mature bone–vascular OLCs with 3D vascular‐like microchannels within 14 days, establishing a verifiable causal loop from molecules to tissue architecture.

### 2.9 Bone–Vascular Organoid‐Like Constructs Accelerate Vascularized Bone Regeneration

To evaluate whether the preorganized osteo–angiogenic architecture of bone–vascular OLCs could be translated into structural bone repair in vivo, rabbits were randomly assigned to three groups and subjected to standardized distal femoral procedures. The Sham group received skin and joint capsule incisions without bone drilling, the Defect group received a cylindrical distal femoral defect measuring 5 mm in diameter and 7 mm in depth, and the bone–vascular OLCs group received the same defect followed by implantation of 7‐day pre‐matured bone–vascular OLCs (Figure S9, Figure 9a). Before implantation, the OLCs had developed a spatially organized vascular‐like network in vitro, providing a prevascularized osteo–angiogenic template for subsequent repair. Because the defect involved both the cortical shell and the marrow‐adjacent cancellous region, repair was assessed by integrating cortical continuity, mineralized bone formation, trabecular remodeling, and vascularized matrix deposition.

**FIGURE 9:**
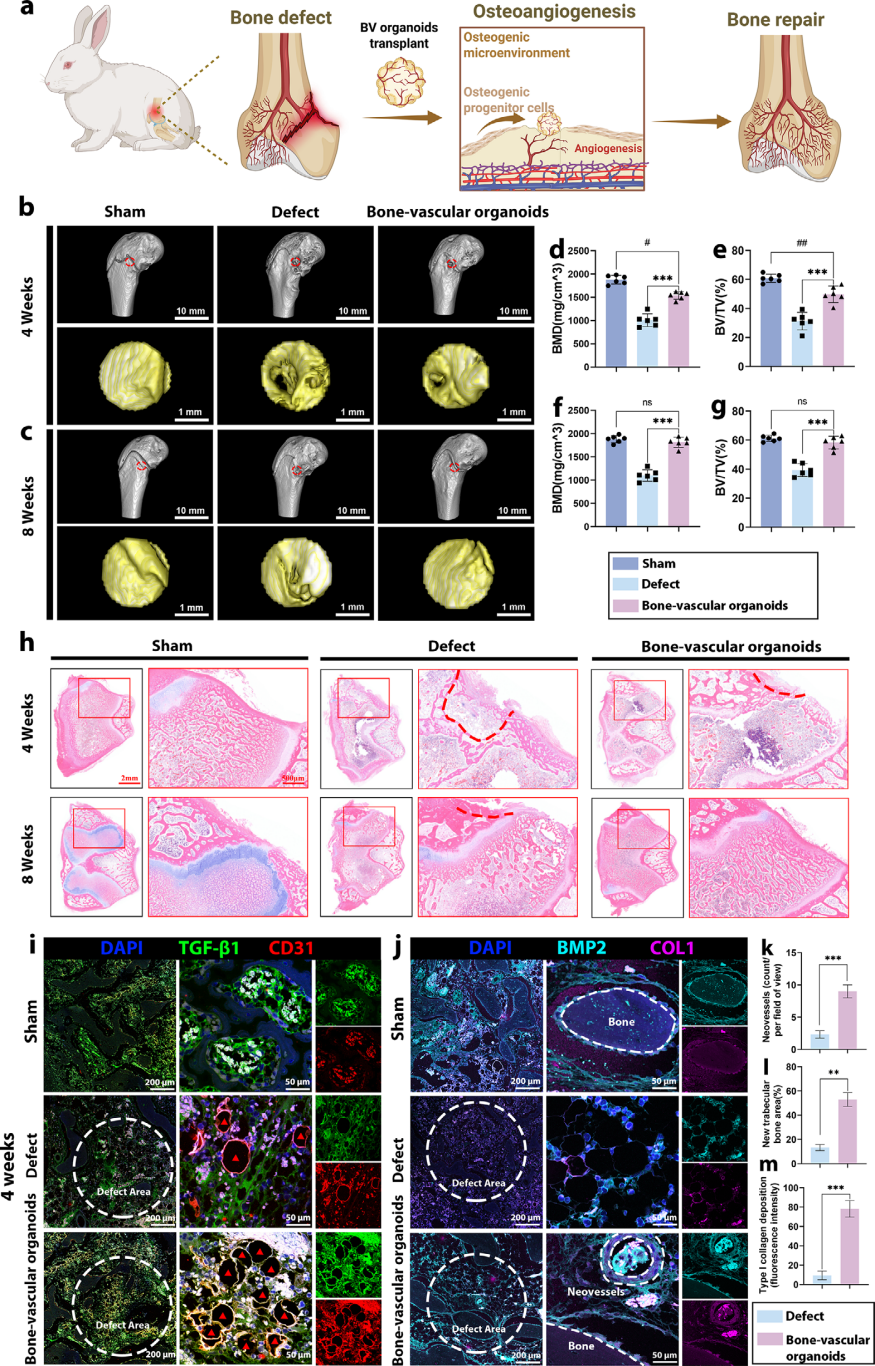
Bone–vascular organoid‐like constructs promote repair of distal femoral defects in rabbits. (a) Schematic illustration of osteo–angiogenic coupling after implantation of bone–vascular OLCs into distal femoral defects. (b, c) Representative micro‐computed tomography (micro‐CT) 3D reconstructions and axial views of distal femoral defects at 4 weeks b) and 8 weeks c) in the Sham, Defect‐only, and bone–vascular OLCs groups. Red circles indicate the defect region or corresponding anatomical region. (d–g) Quantitative micro‐CT analysis of bone mineral density (BMD) and bone volume fraction (BV/TV) at 4 weeks (d, e) and 8 weeks (f, g). n = 6 animals per group. (h) Representative hematoxylin and eosin (H&E) staining at 4 and 8 weeks showing defect bridging, newly formed trabecular bone, and tissue maturation. Boxed regions are shown at higher magnification; dashed outlines indicate the defect area. (i) Immunofluorescence staining of neovascularization within the defect region at 4 weeks in the Defect‐only and bone–vascular OLCs groups. CD31 labels endothelial cells, and TGF‐β1 staining was used to assess growth‐factor‐associated signaling within the defect region. (j) Immunofluorescence staining of newly formed trabecular bone within the defect region at 4 weeks. BMP2 and COL1 indicate osteogenic signaling and collagen matrix deposition, respectively. (k) Quantification of neovessel number within the defect region at 4 weeks, expressed as vessels per high‐power field (HPF; ×640). (l) Quantification of newly formed trabecular bone area within the defect region at 4 weeks. (m) Quantification of COL1 fluorescence intensity from images in j). For k–m, three sections from three defect regions were analysed per animal, with one HPF quantified per section; a randomly selected subset of three animals per group. Data are presented as mean ± s.d. Statistical analyses were performed using one‐way ANOVA with Tukey's multiple‐comparisons test for comparisons among three groups and unpaired two‐tailed Student's t‐test for two‐group comparisons. Scale bars: 10 mm b,c, top; 1 mm b,c, bottom; 2 mm h, low magnification; 500 µm h, high magnification; 200 µm and 50 µm i,j, as indicated. (***P < 0.001; **p < 0.01; *p < 0.05).

Micro‐CT imaging revealed marked differences in defect reconstruction among the three groups. At week 4, 3D reconstructions and axial views showed sparse and irregular mineralized tissue in the Defect group, with persistent discontinuity at the cortical margins (Figure 9b). In contrast, the bone–vascular OLCs group exhibited a more continuous peripheral mineralized band and denser mineralized foci around the defect, indicating accelerated early mineral deposition and partial restoration of cortical continuity. More continuous trabecular structures were also observed on the marrow‐facing side, suggesting concomitant internal reinforcement. By week 8, cavitary defects and structural discontinuities remained evident in the Defect group, whereas the bone–vascular OLCs group displayed a more compact and continuous peripheral architecture approaching the Sham reference morphology (Figure 9c).

Quantitative micro‐CT analysis further supported these observations. At week 4, bone–vascular OLCs implantation markedly increased BMD from approximately 1040 to 1530 mg HA/cm^3^ and BV/TV from approximately 32% to 49% compared with the Defect group, although both indices remained below Sham levels (Figure 9d,e). By week 8, BMD and BV/TV further increased to approximately 1790 mg HA/cm^3^ and 57%, respectively, approaching the Sham reference levels while remaining significantly higher than those in the Defect group, which showed approximately 1100 mg HA/cm^3^ and 39% (Figure 9f,g). These results indicate that bone–vascular OLCs implantation promotes progressive recovery of bone mass and mineralized volume within the defect region, with cortical continuity restoration accompanied by trabecular remodeling.

Histological analysis corroborated the imaging findings. H&E staining showed that the Sham group maintained normal cortical and cancellous architecture at both time points, whereas the Defect group was dominated by fibrous tissue and scattered immature bone at week 4, with limited bridging across the defect margins (Figure 9h). In contrast, the bone–vascular OLCs group showed more abundant new bone formation, early bony connections between defect edges, and increased trabecular filling along the marrow‐facing region. By week 8, the Defect group still retained an obvious inter‐defect gap with immature tissue, whereas the bone–vascular OLCs group exhibited more continuous bone structures traversing the defect, a more compact peripheral bone‐like architecture, and more regularly organized trabecular structures. These histological features indicate that bone–vascular OLCs promote coordinated cortical‐oriented bridging and cancellous‐region remodeling. Histological examination of the heart, liver, spleen, lungs, and kidneys showed preserved tissue architecture without obvious inflammatory infiltration, necrosis, or foreign‐body reactions, supporting the systemic biocompatibility of bone–vascular OLCs implantation (Figure S10).

Given the preorganized vascular‐like vascular architecture of the implanted OLCs, we next examined whether the improved structural repair was accompanied by enhanced angiogenic and osteogenic activity within the defect region. The proposed repair mechanism is illustrated in Figure 9a: BMSC^BMP4+VEGFA^‐derived paracrine cues and bioglass‐derived Ca^2^^+^/Mg^2^^+^ ions provide osteogenic stimulation, while the preformed vascular‐like architecture may facilitate endothelial organization and angiogenic remodeling. At week 4, immunofluorescence staining revealed denser CD31‐positive endothelial structures and stronger TGF‐β1 signals in the bone–vascular OLCs group than in the Defect group, indicating an enhanced pro‐angiogenic microenvironment (Figure 9i). Quantification confirmed a significant increase in neovessel number per high‐power field after bone–vascular OLCs implantation (Figure 9k). In parallel, BMP2 and COL1 staining were markedly enhanced in the bone–vascular OLCs group, indicating increased osteogenic signaling and collagen‐rich matrix deposition within the newly formed bone region (Figure 9j). Consistently, the bone–vascular OLCs group showed a larger newly formed bone area and higher type I collagen fluorescence intensity than the Defect group (Figure 9l,m).

Collectively, these findings demonstrate that bone–vascular OLCs accelerate vascularized bone regeneration in rabbit distal femoral defects. Their therapeutic effect is reflected by early mineral deposition, enhanced angiogenic and osteogenic activity, progressive cortical bridging, and improved trabecular remodeling. Rather than functioning as passive cell aggregates, the pre‐matured bone–vascular OLCs provide a spatially organized osteo–angiogenic template that supports coordinated endothelial organization, collagen‐rich matrix deposition, and structural bone reconstruction. This integrated repair process enabled the bone–vascular OLCs group to approach the Sham reference state by week 8, supporting the potential of prevascularized bone–vascular OLCs for critical‐size bone defect repair.

## 3 Discussion

In this study, we developed a Matrigel‐free living bone graft platform to coordinate osteogenic differentiation, vascular‐like organization, and bone‐like tissue maturation for the repair of large bone defects. By integrating single‐cell‐guided candidate‐cue prioritization, transient paracrine programming of BMSCs, and a GelMA/PEO/BG assembled‐microsphere matrix, we established a development‐informed bone–vascular organoid‐like construct. BMP4 and VEGFA were prioritized as candidate regulators of osteogenesis–angiogenesis coupling, and their combined introduction into BMSCs promoted osteogenic programming, endothelial‐associated sprouting, vascular‐like morphogenesis, extracellular matrix maturation, and bone regeneration in a rabbit distal femoral defect model. The term “development‐informed” does not imply complete reconstruction of the native skeletal developmental niche. Skeletal development is controlled by complex signaling, matrix remodeling, immune regulation, mechanical stimulation, and multicellular interactions. Here, single‐cell transcriptomic information was used to identify actionable osteogenic–angiogenic cues rather than to reproduce the entire developmental program. Accordingly, our platform represents a simplified, function‐oriented approximation that captures selected features of developmental bone formation, including paracrine regulation, osteogenesis–angiogenesis coupling, vascular‐like organization, and matrix maturation. Broader developmental modules, spatial profiling, receptor/pathway blockade, and rescue experiments will be required to improve biological fidelity and establish direct causality. Classical organoids are generally self‐organizing 3D structures derived from stem or progenitor cells that reproduce selected features of tissue development, cellular composition, and organ‐specific architecture. In contrast, the constructs developed here depend on an exogenous microsphere matrix and engineered paracrine cues and therefore do not represent fully self‐organizing classical organoids. We use the term “organoid‐like construct” to describe their multicellular, 3D, function‐oriented organization while explicitly acknowledging their scaffold‐guided engineered nature.

Our findings are consistent with skeletal stem‐cell studies showing that bone formation and repair depend on defined progenitor states and niche‐regulated lineage specification [30]. Chan et al. identified human skeletal stem cells capable of generating bone, cartilage, and stromal lineages, supporting the view that skeletal repair is governed by lineage‐associated programs rather than nonspecific mesenchymal differentiation alone [10, 30, 31]. Because adult bone‐defect environments often lack coordinated biochemical and structural signals, single‐cell information was used here to prioritize BMP4 and VEGFA as candidate osteogenic and angiogenic cues, providing a more rational strategy than conventional empirical induction. Vascular‐like networks can enhance organoid maturation, support vessel formation after transplantation, and improve bone marrow and osteogenic niches [32, 33, 34]. Unlike organoid systems developed primarily for biological modeling, our platform was designed as a transplantable bone–vascular organoid‐like construct that combines organized cellular behavior with therapeutic bone regeneration.

Mechanistically, the regenerative effects of the present system are unlikely to arise from a single dominant pathway. Instead, they appear to reflect the cooperative integration of paracrine, ion‐mediated, matrix‐mediated, and architecture‐mediated cues. Transiently engineered BMSCs function as programmable paracrine units: BMP4 may provide early osteogenic priming, whereas VEGFA may facilitate endothelial‐associated migration, sprouting, and vascular‐like organization. Their complementary activities establish a coordinated osteogenic–angiogenic microenvironment. This interpretation is consistent with the close association of CD31^high^/Emcn^high^ vessels with osteoprogenitors and with evidence that vascular organization and blood flow actively regulate bone formation [35]. Moreover, the biological effects of VEGFA are dose‐dependent, highlighting the importance of controlling the magnitude and duration of angiogenic stimulation during engineered bone regeneration [26]. The transient plasmid‐based strategy used here was therefore intended to provide an early osteogenic–angiogenic signal while reducing the possibility of constitutive long‐term exposure.

In parallel, BG dissolution supplies bioactive ions, including Ca^2^^+^ and Mg^2^^+^, which may reinforce osteogenic differentiation, extracellular matrix production, mineral‐associated maturation, and cell–material interactions [28]. The indirect Transwell experiments support the involvement of diffusible factors because enhanced osteogenic responses were observed without direct contact between recipient cells and engineered constructs. However, these experiments do not fully distinguish the effects of BMP4/VEGFA proteins from those of BG‐derived ionic products. The findings therefore support a combined soluble‐factor mechanism involving both paracrine proteins and bioactive ions. The GelMA/PEO matrix provides a complementary structural and matrix‐mediated context for these soluble signals. GelMA contains cell‐adhesive motifs and supports cell attachment, cytoskeletal organization, extracellular matrix engagement, and matrix deposition, whereas PEO‐mediated phase separation and subsequent leaching generate microcavities and porous topologies that facilitate cell infiltration, mass transport, endothelial invasion, and local matrix organization [19, 21]. This defined Matrigel‐free matrix also improves experimental controllability and translational compatibility by avoiding the tumor‐derived origin, undefined biochemical composition, and batch‐to‐batch variability associated with Matrigel [14, 36]. Therefore, the matrix does not simply serve as a passive carrier, but contributes actively to cell adhesion, osteogenic maturation, vascular permissiveness, and tissue‐scale remodeling.

Together, these findings support a cooperative biomaterial–cell working model. Engineered BMSCs provide lineage‐instructive paracrine signals, BG‐derived Ca^2^^+^ and Mg^2^^+^ provide biochemical reinforcement, GelMA/PEO establishes an adhesive and porous microenvironment, and the assembled‐microsphere architecture spatially integrates these components. Through this multilevel organization, the system supports osteogenic commitment, endothelial‐associated migration, vascular‐like sprouting, extracellular matrix deposition, and mineral‐associated maturation. Because pathway‐specific inhibition, ion‐specific controls, and complete factorial separation of cell‐, ion‐, matrix‐, and architecture‐dependent effects were not performed, the current data do not establish a fully resolved causal network or formal molecular synergy. We therefore interpret the mechanism as a cooperative and integrated effect rather than attributing the outcome to a single dominant pathway.

Compared with recent bone‐organoid and material‐dominant strategies, our platform combines single‐cell‐guided cue prioritization, transient BMSC engineering, bioactive ionic stimulation, and a Matrigel‐free microstructured matrix. Previous studies have generated vascularized and mineralized bone organoids or ossification‐center‐like constructs for bone regeneration [31, 37]. In parallel, oriented cortical‐bone‐like lamellar scaffolds, hyperelastic bone scaffolds, and mechanically conditioned tissue‐engineered grafts have emphasized architectural guidance, scalable fabrication, mechanical strength, and mechanically regulated maturation [38, 39, 40]. Our approach instead emphasizes biological programmability: engineered cells provide development‐associated paracrine instructions, whereas the material niche provides defined biochemical and spatial support for self‐organizing bone–vascular tissue formation. Nevertheless, this comparison also highlights an important limitation. Although the current platform provides strong biological instructiveness, its mechanical properties remain insufficient for direct repair of load‐bearing segmental defects. Furthermore, although early canal‐like microarchitectures were observed, additional structural and functional analyses are required before these features can be considered equivalent to mature osteonal structures or fully perfusable vascular networks.

Several limitations of the present study should be acknowledged. First, BMP4 and VEGFA represent only selected components of the complex native skeletal niche, and the individual contributions of engineered paracrine proteins, BG‐derived ionic products, matrix‐mediated adhesion, and assembled architecture were not completely separated. Future studies incorporating pathway‐specific inhibitors, neutralizing antibodies, ion‐matched controls, BG‐free matrices, and architecture‐matched comparisons will be required to clarify the relative contribution of each component. Second, although the transient plasmid‐based strategy was designed to minimize prolonged exposure to potent osteogenic and angiogenic signals, comprehensive long‐term safety evaluations remain necessary. Potential risks, including ectopic mineralization or ossification, abnormal vascularization, immune responses, and uncontrolled cytokine release, should be systematically assessed in future studies. Third, osteoclast‐mediated remodeling was not incorporated, which may limit the recapitulation of long‐term bone turnover and lamellar remodeling [41]. Fully perfusable and hierarchically organized vascular networks with mural‐cell or smooth‐muscle stabilization were also not achieved, and further integration of functional perfusable vascular architectures will be required [42]. Fourth, the mechanical properties of the constructs remain insufficient for direct application in load‐bearing defects. Incorporating osteoclast regulation, immune and neural cues, perfusable vascular networks, mechanically reinforced architectures, and dynamic mechanical stimulation may improve physiological relevance [43, 44]. Finally, validation in large‐animal, load‐bearing segmental defect models and clinically relevant implantation scenarios will be essential to evaluate long‐term integration, mechanical adaptability, biosafety, manufacturing scalability, and translational feasibility.

Overall, this study demonstrates that developmental information can guide the design of programmable bone–vascular organoid‐like constructs. Rather than reproducing skeletal development in its entirety, the platform integrates engineered paracrine signaling, bioactive ionic stimulation, matrix‐mediated regulation, and assembled‐microsphere architecture within a simplified and controllable system. This cooperative design provides a potential framework for constructing vascularized living bone grafts and highlights the importance of coordinating cellular instructions with biomaterial‐defined biochemical and spatial microenvironments.

## 4 Conclusions

In summary, this study establishes a development‐informed, Matrigel‐free, and spatiotemporally programmed bone–vascular organoid‐like platform for large bone defect repair. By integrating single‐cell‐guided prioritization of BMP4/VEGFA‐associated cues, transient BMSC paracrine programming, and a defined GelMA/PEO/BG assembled‐microsphere matrix, we achieved the sequential construction of osteoinductive bone OLCs and vascularized bone–vascular OLCs with organized matrix deposition and vascular‐like microarchitectures. Rather than fully reproducing skeletal development or classical organoid self‐organization, this platform combines selected development‐associated cellular cues with biomaterial‐defined biochemical and spatial regulation. These findings support the potential of Matrigel‐free bone–vascular OLCs as programmable living grafts for vascularized bone regeneration.

## 5 Experimental Section

### 5.1 Single‐Cell RNA‐seq Data Analysis

Single‐cell RNA sequencing (scRNA‐seq) data were retrieved from the GEO database under accession number GSE156635, comprising four biological samples (BSC1–BSC4). Initial processing of the count matrices was conducted using the Seurat (v5.4.0) R package. Quality control (QC) was performed by retaining cells expressing at least 200 genes and genes detected in at least 3 cells; mitochondrial gene content was calculated via the PercentageFeatureSet function to evaluate cell viability. The data were normalized using a global‐scaling method (LogNormalize), and the top 2,000 highly variable genes were identified using the variance‐stabilizing transformation (VST) method. To mitigate batch effects across the four samples, an anchor‐based integration strategy was implemented based on the first 20 principal components (PCs), followed by data scaling. Unsupervised clustering was performed using a Shared Nearest Neighbor (SNN) graph‐based approach on the integrated space, and the resulting clusters were visualized using t‐SNE for non‐linear dimensionality reduction. For the specific comparison between BMSCs and other cell lineages, a more stringent threshold of P.adj< 0.05, |avg_log2FC| > 1. Finally, Gene Ontology (GO) enrichment analysis for the biological process (BP) category was conducted using the clusterProfiler package and the org.Mm.eg.db database. Multiple testing corrections were performed using the Benjamini method to elucidate the functional role of BMSCs in bone‐vessel coupling.

### 5.2 hdWGCNA Analysis

hdWGCNA was performed on the annotated single‐cell dataset to identify BMSC‐associated co‐expression modules. Cell‐type labels were standardized, and metacells were generated according to cell type and sample identity. Highly variable genes were used to construct a signed co‐expression network. Module eigengenes were calculated and correlated with cell‐type identities. The blue module showed the strongest association with BMSCs and was selected as the BMSC‐associated module for downstream analysis.

### 5.3 Functional Candidate Screening

To identify functionally relevant BMSC‐associated genes, BMSC upregulated DEGs were intersected with genes from the hdWGCNA blue module and GO term‐associated gene sets. Two functional axes were analyzed separately. The osteogenic axis used the bone mineralization GO term, whereas the angiogenic/endothelial axis used the endothelial cell differentiation GO term.

### 5.4 Cell‐Cell Communication Analysis

BMSC‐to‐endothelial communication was evaluated using CellChat and NicheNet. CellChat was used to infer ligand‐receptor communication probability, whereas NicheNet was used to estimate ligand activity associated with endothelial transcriptional responses. Ligand‐level evidence from the two methods was calibrated using shared ligands detected by both methods and integrated into a relative ligand‐prioritization score. Ligands detected by both methods were used as anchors for score calibration, and ligand‐level evidence was integrated into a ligand‐prioritization score for ranking functional candidate genes.

### 5.5 Synthesis of GelMA

Porcine skin gelatin (50 g; Sigma‐Aldrich, Shanghai, China) was dissolved in phosphate‐buffered saline (PBS, pH 7.4; Viva Cell, Shanghai, China) to a final volume of 500 mL at 37°C under constant stirring. Methacrylic anhydride (MAA, 10 mL; Sigma‐Aldrich, Shanghai, China) was added dropwise, and the mixture was allowed to react for 3 h at 37°C. The reaction mixture was transferred to dialysis bag (MWCO 8–10 kDa, Solarbio, Beijing) and dialyzed against deionized water at 37°C for 4 Day with daily water changes. The retentate was then lyophilized to yield GelMA as a white, porous foam.

### 5.6 Nuclear Magnetic Resonance Spectroscopy Analysis

Gelatin and methacrylated gelatin (GelMA) samples were dissolved in 600 µL of deuterium oxide (D_2_O). ^1H nuclear^ magnetic resonance spectra were acquired at 400 MHz using an NMR spectrometer (AVANCE III 400, Bruker, Germany) at 25°C. For GelMA, the degree of methacrylation (DoM) was quantified by integrating the methacrylate vinyl proton signals in the range of 5.3–6.1 ppm (*I_vinyl_*) and normalizing them to the gelatin α‐CH proton signals at 3.9–4.5 ppm (*I_α‐CH_*). The DoM was calculated according to Equation (1):

(1) $$\;\mathrm{Degree}\;\mathrm{of}\;\mathrm{Methacrylation}\left(\%\right)=\frac{I_{vinyl}/n_{vinyl}}{I_{\alpha -CH}/n_{\alpha -CH}}$$

*I*_vinyl_: is integration of the allyl –CH═CH_2_ protons (5.3–6.1 ppm), *n*_vinyl_ is number of vinyl protons per MAA unit (═3; two –CH_2_ + one –CH), *I*_α‐CH_ is integration of gelatin α‐CH protons (3.9–4.5 ppm) and *n*_α‐CH_ is number of α‐CH protons per amino acid residue (═1). As calculated, the GelMA synthesized by this method has a degree of methacrylation of approximately 40%. All integrations were performed using MestReNova software (Version 14.0, Mestrelab Research S.L., Spain) with automatic phase and baseline correction. For each material, 1H NMR spectra were recorded from independently synthesized batches (n = 3). The DoM was calculated for each batch as described above, and the results are reported as mean ± standard deviation (SD). (In Figure S2a)

### 5.7 Fourier Transform Infrared Spectroscopy Analysis

Fourier transform infrared (FTIR) spectroscopy was employed to characterize gelatin and methacrylated gelatin (GelMA) before and after methacrylation. Lyophilized samples were analyzed using an FTIR spectrometer (Nicolet iS50, Thermo Fisher Scientific, USA) equipped with an attenuated total reflectance (ATR) accessory at room temperature. Spectra were collected over the wavenumber range of 4000–500 cm^−^^1^ with a spectral resolution of 4 cm^−^^1^, and 32 scans were averaged for each measurement. Successful methacrylation of gelatin was confirmed by the appearance of characteristic methacrylate‐associated absorption bands in the GelMA spectra, including the C═C stretching vibration at approximately 1635–1640 cm^−^^1^ and the C–H bending vibrations of methacrylate groups at around 1450–1470 cm^−^^1^, which were absent in native gelatin. In addition, the ester carbonyl (C═O) stretching peak at approximately 1720 cm^−^^1^ further indicated the introduction of methacrylate moieties. The characteristic amide I (∼1650 cm^−^^1^), amide II (∼1540 cm^−^^1^), and amide III (∼1240 cm^−^^1^) bands of gelatin were retained after methacrylation, confirming preservation of the gelatin backbone. All spectra were processed using OMNIC software (Version 9.2, Thermo Fisher Scientific, USA) with automatic baseline correction and normalization. FTIR measurements were performed on independently synthesized batches for each material (n = 3), and representative spectra are presented (Figure S2b).

### 5.8 Swelling and Degradation Analysis

Hydrogel discs were prepared in four formulations–Control (GelMA), BG (GelMA + bioactive glass), PEO (GelMA + PEO), and PEO/BG (GelMA + PEO + bioactive glass). For swelling, each specimen was gently blotted and weighed to obtain its initial mass *W*_0_(n = 5 per formulation), then immersed in PBS at 37°C. PBS was refreshed every 2 days. At predefined intervals, samples were removed, the surface moisture was carefully wicked away with lint‐free wipes, and the wet mass *W_t_* was recorded to generate time‐dependent swelling profiles until equilibrium was reached. The swelling ratio was calculated as

$$\mathrm{Swelling}\;\mathrm{ratio}\;(\%)=\frac{W_{t}-W_{0}}{W_{0}}\times 100$$

Time‐course swelling curves for all four formulations are reported in Figure S2c.

### 5.9 Degradation Performance (Mass‐Loss Method)

Disc‐shaped hydrogels for each formulation–Control (GelMA), BG (GelMA + bioactive glass), PEO (GelMA + PEO), and PEO/BG (GelMA + PEO + bioactive glass).; diameter 8 mm, thickness ∼2 mm) were photo‐crosslinked as described above and rinsed three times with 1× PBS to remove unreacted species. Samples were then frozen at −80°C and lyophilized to constant mass (≤ 5% variation over 12 h). The initial dry mass *M*_0_ was recorded on an analytical balance (readability 0.1 mg). For each group (n = 5), specimens were incubated individually in 10 mL of 1× PBS (pH 7.4) at 37°C under gentle agitation (∼60 rpm), with PBS refreshed every 48 h. At predefined time points (Day 0, 1, 3, 7, 10, and 14), samples were removed, briefly rinsed with deionized water to eliminate residual salts, gently blotted to remove surface liquid, re‐frozen (−80°C), and lyophilized to constant mass to obtain *M_i_*. The degradation rate (%) was calculated as:

$$\mathrm{Degradation}\;\mathrm{rate}\;(\%)=\frac{M_{0}-M_{i}}{M_{0}}\times 100$$

Results are reported as mean ± SD, and a degradation profile from Day 0–14 was plotted with the y‐axis representing degradation rate (%) and error bars indicating SD (Figure S2d). Consistent with the accompanying swelling/mass‐change trends in the same batch, rapid early changes (Day 1‐3) were followed by near‐plateaus; among formulations, GBG and GPBG exhibited lower swelling and slower mass loss than G and GP.

### 5.10 Synthesis and Elemental Characterization of Bioactive Glass

Bioactive glass (BG) particles composed of Na_2_O, K_2_O, MgO, CaO, and SiO_2_ were prepared by a melt‐quenching method. Briefly, analytical‐grade sodium carbonate, potassium carbonate, magnesium oxide, calcium carbonate, and silicon dioxide were weighed at a molar ratio of 1:1:1:1:9 and thoroughly mixed using a ball mill to obtain a homogeneous precursor powder. The mixed powder was transferred into an alumina crucible and heated in a muffle furnace at 1100°C for 5 h until a transparent and bubble‐free glass melt was obtained. The melt was then rapidly quenched to room temperature to form bulk glass. The as‐quenched glass was crushed, ground, and sieved using a ball mill to obtain BG particles with the desired particle‐size range. The resulting powders were dried and stored in a desiccator before further use.

The morphology and elemental distribution of the BG particles were characterized by field‐emission scanning electron microscopy equipped with an Oxford Instruments energy‐dispersive X‐ray spectroscopy system (SEM4000X, CIQTEK, China). Before imaging, the BG powders were uniformly dispersed onto conductive carbon tape and sputter‐coated with a 10 nm platinum layer to improve surface conductivity. Elemental mapping, point‐scan, and area‐scan analyses were performed to verify the presence and spatial distribution of Na, K, Mg, Ca, Si, and O within the BG particles. The EDS signals were used to confirm the successful incorporation of the designed oxide components and the compositional homogeneity of the prepared BG particles.

### 5.11 Rheological Properties of Hydrogels

Hydrogels were photocured in situ on an HR‐20 rheometer (TA Instruments, USA) equipped with a 20‐mm parallel‐plate geometry and a solvent trap. Precursor solutions were loaded at 37°C (gap, 1.0 mm), allowed to level for 2 min, and continuously irradiated at 405 nm until the storage modulus (G′) reached a plateau. After curing, samples were equilibrated at 37°C for 5 min and subjected to time‐sweep measurements under small‐amplitude oscillation (strain 1% within the linear viscoelastic region, LVR; angular frequency 10 rad s^−^^1^) to record G′ and the loss modulus (G″) as functions of time (n = 3). Upon completion of the time sweep, the same cured specimens were tested at 37°C in oscillatory mode at a fixed strain within the LVR (typically 0.5–1.0%, predetermined by amplitude sweeps; see panel h). The angular frequency was swept from 0.1 to 100 rad s^−^^1^, and G′, G″, and the loss tangent tan δ (= G″/G′) were recorded to evaluate the viscoelastic spectrum and elasticity dominance (n = 3). For pre‐photocuring characterization, uncured precursor inks were loaded at 37°C (gap, 1.0 mm) under a solvent trap to minimize evaporation. Using a strain within the LVR established for each formulation (see panel h), angular frequency was swept from 0.1 to 100 rad s^−^^1^. G′, G″, and tan δ were collected to assess viscoelastic properties pertinent to printability and flow (n = 3). No light exposure was applied during these measurements. To define the LVR and yield point for each precursor, amplitude (strain) sweeps were performed at 37°C at a fixed angular frequency of 10 rad s^−^^1^. Strain was increased logarithmically from 0.01% to 100% (or until G′ deviated by >5% from its plateau). The LVR limit was defined as the highest strain at which G′ remained constant; all subsequent small‐amplitude oscillatory tests (panels e–g) were conducted at strains below this limit (n = 3). Before all oscillatory tests, the normal force was adjusted to approximately 0 N to avoid over‐compression, and sample edges were trimmed after loading. Photocuring followed a continuous 405 nm protocol (time sweep at 1% strain and 10 rad s^−^^1^ until G′ plateaued), after which post‐cure tests were carried out at 37°C.

### 5.12 Cell Culture

Rabbit umbilical vein endothelial cells (RUVECs), rabbit bone‐marrow–derived mesenchymal stem cells (BMSCs, Figure S3) and rabbit osteoblastic progenitor cells (VivaCell, Shanghai, China) were expanded in Dulbecco's modified Eagle's medium/Nutrient Mixture F‐12 (DMEM/F‐12; VivaCell, Shanghai, China) supplemented with 10% (v/v) fetal bovine serum (FBS; VivaCell) and 1% (v/v) penicillin–streptomycin (10 000 U mL^−^^1^ penicillin, 10 mg mL^−^^1^ streptomycin; VivaCell). Cultures were maintained at 37°C in a humidified 5% CO_2_ atmosphere, and the growth medium was replaced every 24 h. For osteogenic induction, subconfluent rBMSCs were transferred to osteogenic differentiation medium consisting of α‐MEM (Procell, Wuhan, China) supplemented with 10 mM β‐glycerophosphate, 50 µg mL^−^^1^ L‐ascorbic acid, 100 nM dexamethasone, 5% FBS, and 1% penicillin–streptomycin. Cells were cultured for up to 14 d with medium renewal every 2 day.

### 5.13 Fabrication of PEO/BG aMS

PEO/BG aMS were produced with a water‐in‐oil microfluidic approach and subsequently assembled into clusters (Figure 1a). In brief, GelMA (10 wt.%) and polyethylene oxide (PEO, 0.5 wt.%) were dissolved in sterile phosphate‐buffered saline (PBS) under gentle magnetic stirring until a homogeneous solution was obtained. Bioactive glass 1393 powder (< 50 µm, 2 mg/mL) and lithium phenyl‐2,4,6‐trimethylbenzoylphosphinate (LAP, 0.3 wt.%) were then added and dispersed uniformly to yield the hydrogel precursor. The aqueous precursor was injected into a polydimethylsiloxane flow‐focusing microfluidic chip, while sterile mineral oil acted as the continuous phase, generating monodisperse hydrogel droplets by shear‐induced breakup. The freshly collected droplets were transferred into 1.5 mL Eppendorf tubes pre‐filled with mineral oil; capillary forces drove their spontaneous close‐packing into microsphere clusters. The clusters were photo‐crosslinked under 405 nm light (10 mW cm^−^^2^, 30 s). After curing, the constructs were washed three times with sterile PBS to remove residual oil. Incubation at 37°C enabled sacrificial extraction of the PEO porogen, producing an interconnected macroporous architecture within each microsphere. The resulting PEO/BG aMS clusters were stored in PBS at 4°C and used within 48 h for subsequent experiments.

### 5.14 Transwell‐Mediated Indirect Osteogenesis Assay

BMP4‐overexpressing bone‐marrow‐derived mesenchymal stem cells (^BMSCBMP4^) were seeded into the pores (1 × 10^6^ cells g^−^^1^ microsphere) and allowed to adhere for 6 h before use. Osteoprogenitor cells were plated in the bottom chamber of 12‐well Transwell plates (5 × 10^4^ cells well^−^^1^, growth medium). After 24 h the inserts (0.4 µm pore size) were loaded with one of four formulations (100 mg): BMSC (PEO aMS+ BMSC, no stimulus), BMSC^BMP4^ (PEO aMS+BMSC^BMP4^, paracrine only), BMSC/BG (PEO/BG aMS+ BMSC, ionic release only), and BMSC^BMP4^/BG (PEO/BG aMS+BMSC^BMP4^, combined paracrine + ionic release). Cultures were switched to osteogenic medium (α‐MEM, 10% FBS, 50 µg mL^−^^1^ ascorbic acid, 10 mM β‐glycerophosphate, 100 nM dexamethasone) and maintained for 14 d with medium renewal every other day. Alizarin red S (ARS) and alkaline phosphatase (ALP) staining were carried out on day 7. Stained areas were quantified with ImageJ. Immunofluorescence for BMP2, osteocalcin (OCN) and Runx2 (nuclear) was performed (primary antibodies 1:200, overnight 4°C; Alexa Fluor 488 secondary 1:400, 1 h). Mean fluorescence intensity (MFI) of ten random fields was analyzed using the Fiji “Measure” plugin.

### 5.15 Rabbit Distal Femoral Defect Model

Animal experiments were approved by the Experimental Animal Welfare and Ethics Committee of the Laboratory Animal Center of Ningbo University under approval No. NBU20250390. Thirty‐six adult female New Zealand White rabbits, approximately 3 months old and weighing 2.5 ± 0.5 kg, were obtained from Fumaikang Laboratory Animal Technology Co., Ltd. The animals were acclimatized for 7 days before surgery and randomly allocated to the Sham, Defect, and bone–vascular OLCs groups (n = 12 per group). Within each group, six animals were euthanized at 4 weeks and the remaining six at 8 weeks after surgery, resulting in a sample size of n = 6 per group at each time point.

After fasting for 12 h, the rabbits were anesthetized with inhaled isoflurane at approximately 4% for induction and 2% for maintenance. Through a lateral surgical approach, the distal femur was exposed, and the periosteum was carefully reflected to visualize the lateral femoral condyle. In the Sham group, the skin and joint capsule were incised and the lateral femoral condyle was exposed without bone drilling or construct implantation. In the Defect group, a cylindrical defect measuring 5 mm in diameter and 7 mm in depth was created using a low‐speed surgical drill under continuous sterile saline irrigation and was left empty. In the bone–vascular OLCs group, an identical defect was created and immediately filled with seven‐day‐matured bone–vascular OLCs composed of PEO/BG aMS seeded with BMSC^BMP4+VEGFA^. Following implantation, the periosteum and skin were closed separately using 4‐0 absorbable sutures. Postoperative care included intramuscular administration of penicillin at 40 000 IU kg^−^^1^ day^−^^1^ for three consecutive days. The animals were monitored daily for body temperature, food intake, activity, wound healing, and signs of postoperative complications. The experimental timeline and surgical procedures are schematically summarized in Figure S9. At 4 and 8 weeks after surgery, the animals were euthanized, and the distal femoral specimens were harvested. Bone regeneration was evaluated using micro‐computed tomography at a voxel size of 20 µm. Standardized regions of interest corresponding to the original defect area were used to quantify new bone formation and trabecular architecture. The specimens were subsequently fixed, decalcified, embedded in paraffin, sectioned, and stained with hematoxylin and eosin for histological evaluation of defect bridging, new bone formation, and tissue organization. Because the surgeon was required to perform group‐specific procedures, blinding during surgery was not feasible. After specimen collection, all samples and imaging datasets were assigned coded identifiers by an investigator who was not involved in outcome assessment. Micro‐CT reconstruction, region‐of‐interest selection, quantitative measurements, and histological evaluation were subsequently performed by investigators blinded to group allocation.

### 5.16 Isolation and Culture of Rabbit Bone‐Marrow‐Derived Mesenchymal Stem Cells

Healthy New Zealand White rabbits (2 weeks) were anesthetized with 3% isoflurane for 10 min, both hind limbs were shaved, disinfected with 75% ethanol, and expose the femora. The greater trochanter and lateral condyle were excised aseptically, and bone marrow was flushed from each femoral shaft with complete DMEM/F‐12 (10% premium FBS, 1% penicillin‐streptomycin) using a 20‐mL syringe; the suspension was diluted 1: 3 with DMEM/F‐12 medium, filtered through a 100 µm strainer, and seeded into T‐75 flasks at 1×10^4^‐1×10^5^ nucleated cells per 75 cm^2^ in 15 mL medium. Cells were maintained at 37°C, 5% CO_2_, with half‐medium replacement at 48 h to remove non‐adherent cells and complete medium changes every 2 d thereafter. When confluence reached 70–80%, adherent cells were detached with 0.25% trypsin–EDTA, centrifuged (1000 × g, 5 min), and replated at a 1:3 ratio. ells were phenotypically confirmed by flow cytometry (CD44^+^/CD90^+^, CD14^−^/CD34^−^) and tri‐lineage (osteogenic, adipogenic, chondrogenic) differentiation to verify mesenchymal multipotency. (In Figure S3)

### 5.17 Construction and Validation of BMSC^BMP4+VEGFA^

Rabbit bone‐marrow mesenchymal stem cells (passages 3–5) were seeded at 5 × 10^4^ cells per well in 24‐well plates with complete DMEM/F‐12. After reaching 70–80% confluence, they were transfected overnight under serum‐free conditions using a plasmid mixture of pCMV‐BMP4(rabbit)‐3×FLAG‐Neo and pCMV‐VEGFA(rabbit)‐3×FLAG‐Neo (0.5 µg each, total 1 µg), complexed for 15 min at 25°C with 2 µL Lipo8000 in 100 µL serum‐free DMEM/F‐12 (plasmid DNA:Lipo8000 = 0.5 µg:2 µL). After 12 h, the transfection medium was replaced with fresh complete DMEM/F‐12 (1500 µL), and cells were cultured for an additional 24–72 h before analysis. Expression of target genes and proteins was confirmed by quantitative RT‐PCR (SYBR Green, GAPDH reference) at 24 h, immunofluorescence (4% paraformaldehyde fixation, anti‐BMP4, anti‐VEGFA; Nikon AXR with NSPARC confocal microscopy) at 48 h, and Western blotting (anti‐BMP4, anti‐VEGFA, anti‐β‐actin as control) at 72 h. (In Figure 2) And plasmid maps of the overexpression constructs were shown in Figure S4.

### 5.18 Enzyme‐Linked Immunosorbent Assay (ELISA)

Conditioned media were harvested from each culture, cell debris was cleared by centrifugation (500 × g, 5 min, 20 °C) and kept on ice until analysis. Rabbit‑specific sandwich ELISA kits (Vascular Endothelial cell Growth Factor/VEGF, CSB‑E08524Rb, CUSA Bio; Bone Morphogenetic Protein‐4/BMP‑4, YJ31326, Bone Morphogenetic Protein‐2/BMP‑2, YJ31327, Fibroblast Growth Factor‐2/FGF‑2, YJ31408, Platelet Derived Growth Factor‐BB/ PDGF‑BB, YJ31398, Platelet Derived Growth Factor‐AA/PDGF‑AA, YJ31424, C‐X‐C Motif Chemokine Ligand 12/CXCL12, YJ31180, all Yuanju Bio, China) were equilibrated to room temperature for 20 min before use. A two‑fold serial dilution of the supplied standard in 250 µL volumes produced concentrations of 3000, 1500, 750, 375, 187.5, 93.75 and 46.875 pg mL^−^^1^; buffer alone served as the 0 pg mL^−^^1^ blank. One hundred microlitres of each standard or clarified sample were loaded in duplicate into pre‑coated 96‑well plates and incubated for 120 min at room temperature. Plates were then washed five times with wash buffer, blot‑dried on absorbent paper, and incubated for 60 min with 100 µL well^−^^1^ biotin‑conjugated detection antibody. After a second five‐cycle wash, 100 µL well^−^^1^ horseradish‑peroxidase conjugate were added and allowed to react for 20 min in the dark. Following another five washes, color was developed with 100 µL well^−^^1^ TMB substrate for 15–20 min until the highest standard exhibited a pronounced color change. Reactions were stopped with 50 µL well^−^^1^ stop solution, and absorbance was read immediately at 450 nm using a microplate spectrophotometer (Thermo Fisher, America). Cytokine concentrations were calculated from a four‑parameter logistic standard curve generated from the duplicate standards, and mean values from duplicate sample wells were used for subsequent statistical analyses.

### 5.19 Micro‐CT Evaluation

Tissues from all experimental New Zealand White rabbits were harvested at 4 and 8 weeks postoperatively, fixed in 4% paraformaldehyde for 48 h at room temperature, and rinsed with PBS. Rabbit femoral specimens were then scanned using a micro‐CT system (Duper Nova; PINGSENG Healthcare Inc., China). 3D reconstructions were generated with the manufacturer's analysis software, and morphometric indices—including bone volume fraction (BV/TV) and bone mineral density (BMD)—were quantified within standardized regions of interest (ROIs).

### 5.20 Quantitative Real‐Time PCR (qRT‐PCR)

After plasmid transfection, total RNA was isolated from BMSCs using TRIzol Reagent (TransGen, China) according to the manufacturer's instructions. The purified RNA was reverse‐transcribed into cDNA with the EasyScript All‐in‐One First‐Strand cDNA Synthesis SuperMix for qPCR (TransGen, China). Quantitative real‐time PCR was performed on a LightCycler 480 Instrument II (Roche, Switzerland) using PerfectStart Green qPCR SuperMix (TransGen, China). Relative mRNA levels were calculated by the 2^−ΔΔCt^ method, with GAPDH serving as the internal reference gene. Primer sequences are provided in Table S1. For each experimental group, three biological replicates were analyzed.

### 5.21 RNA Sequencing (RNA‐seq) and Bioinformatic Analyses

Two OLCs types were examined—bone OLCs and bone–vascular OLCs. After 14 days of culture, constructs were incubated in a GelMA dissolution/lysis buffer (EFL‐GM‐LS‐001, Engineering For Life, China) to release encapsulated cells, then centrifuged to collect cell pellets for downstream processing. Total RNA was isolated and purified with TRIzol reagent (TransGen Biotech, China) according to the manufacturer's protocol. RNA‐seq libraries were prepared and sequenced by OE Biotech Co., Ltd. (Shanghai, China) on an Illumina NovaSeq 6000 platform. All sequencing reads have been deposited in the National Center for Biotechnology Information (NCBI) Sequence Read Archive (SRA). For bioinformatics, raw reads underwent quality control and adapter/quality trimming, followed by alignment or quasi‐mapping to the appropriate reference transcriptome and transcript quantification. Gene‐level counts were derived and normalized; differential expression between groups was tested with false‐discovery‐rate correction (Benjamini–Hochberg). Functional enrichment analyses (GO and KEGG) were performed on significantly regulated genes to identify over‐represented biological processes and pathways.

### 5.22 siRNA‐Mediated Knockdown of BMP4 and VEGFA

Three independent siRNA sequences targeting *BMP4* (si‐BMP4‐17/27/63) and three targeting *VEGFA* (si‐VEGFA‐743/852/1003) were designed and synthesized (Sangon Biotech, China). A non‐targeting siRNA (siNC) was included as a negative control. Preliminary experiments were conducted to identify the siRNA sequence with the highest knockdown efficiency for each gene (Table S2).

BMSCs were seeded in six‐well plates and cultured to 60–70% confluence. Prior to transfection, culture medium was removed, and cells were gently washed 1–2 times with PBS. For each well, 800–1000 µL Opti‐MEM (Thermo Fisher, 31985070, America) was added, and cells were pre‐incubated at 37°C for 30 min. For each well, 250 µL Opti‐MEM containing 5 µL Lipofectamine Stem Transfection Reagent (Thermo Fisher, STEM00001, America) was combined with 250 µL Opti‐MEM containing 5 µL siRNA (siNC or gene‐specific siRNA) and gently mixed. The mixture was incubated at room temperature for 20 min to allow formation of stable siRNA‐lipid complexes. The siRNA‐lipid complex was added dropwise to cells. After 6–8 h, the medium was replaced with fresh complete growth medium. Cells were further cultured for 24–48 h prior to harvest. Total RNA was extracted using standard protocols, and knockdown efficiency was evaluated by qRT‐PCR. The siRNA sequence achieving the highest knockdown efficiency was selected for downstream functional experiments. BMSCs were seeded and transfected as described above. Experimental groups included untreated control (NC), Negative control siRNA (siNC), and gene‐specific siRNA (si‐BMP4 or si‐VEGFA). After 6–8 h transfection, culture medium was replaced with fresh complete medium. Cells were further cultured for 24–48 h, after which total RNA was collected for qRT‐PCR analysis. After testing, si‐BMP4‐27 and si‐VEGFA‐852 showed the best effect of gene knockdown.

### 5.23 Western Blotting

Cells and OLCs were lysed in RIPA buffer supplemented with protease and phosphatase inhibitors. Lysates were cleared by centrifugation (12,000 rpm, 15 min, 4°C), and protein concentrations were determined by BCA assay. Equal amounts of protein (20 µg per well) were resolved by SDS–PAGE and transferred to 0.45µm PVDF membranes. Membranes were blocked with 5% (w/v) nonfat milk in TBST (0.1% Tween‐20) for 1 h at room temperature and incubated with primary antibodies overnight at 4°C, followed by HRP‐conjugated secondary antibodies for 1 h at room temperature. The primary antibodies used included VEGFA (Affinity, AF5131, 1:1000), RUNX2 (Affinity, AF5186, 1:1000), BMP2 (Proteintech, Ag13501, 1:1000), BMP4 (Proteintech, 12492‐1‐AP, 1:1000), TGF‐β1 (Proteintech, Ag13591, 1:1000), Collagen Type I (Proteintech, 67288‐1‐Ig, 1:1000), WNT2 (Proteintech, 66656‐1‐Ig, 1:1000), WNT3A (Proteintech, Ag25051, 1:1000), Beta Actin (Proteintech, 66009‐1‐Ig, 1:1000), GAPDH (Proteintech, Ag0766, 1:1000) and the secondary antibodies used included HRP‐conjugated Goat Anti‐Rabbit IgG (Proteintech, SA00001‐2, 1:5000) and HRP‐conjugated Goat Anti‐Mouse IgG (Proteintech, SA00001‐1, 1:5000). Signals were detected by ECL and imaged on a chemiluminescence system (ChemiDoc Imaging System, Bio‐RAD, America). Densitometry was performed in ImageJ; target proteins were normalized to total protein or housekeeping controls as indicated. For phospho‐proteins, total protein blots were run and quantified in parallel. Uncropped blots are provided in Figure S6.

### 5.24 Immunofluorescence Staining of OLCs

OLCs were cultured in 2% (w/v) low‐concentration GelMA hydrogel within DMEM/F12 medium. On days 3, 5, 7, and 14, the peripheral GelMA surrounding the OLCs was gently rinsed with PBS. Samples were then fixed at 4°C for 8 h in pre‐chilled 4% paraformaldehyde plus 0.05% glutaraldehyde (in PBS, pH 7.4), followed by permeabilization with 0.5% Triton X‐100 for 2 h. Afterward, specimens were blocked with 3% BSA for 3 h and incubated with fluorophore‐conjugated primary antibodies at 4°C for 8 h. Unbound primary antibodies were removed, after which samples were stained with TRITC‐phalloidin (Solarbio, CA1610; 1:200) and counterstained with DAPI. OLCs were maintained in 0.5% sodium alginate solution and imaged on a confocal microscope using a 10× water‐immersion objective (Nikon AXR with NSPARC). Quantitative analysis of immunofluorescence staining was performed using ImageJ. The primary antibodies used included: CoraLite Plus 488‐conjugated BMP2 monoclonal antibody (Proteintech, 1:200, 66383‐1‐Ig); CoraLite Plus 488‐conjugated osteocalcin/OCN polyclonal antibody (Proteintech, 1:200, 23418‐1‐AP); CoraLite Plus 488‐conjugated CD31 monoclonal antibody (Proteintech, 1:200, CL488‐66065); CoraLite 647‐conjugated VEGFA monoclonal antibody (Proteintech, 1:200, 66828‐1‐Ig); CoraLite 488‐conjugated VEGFA monoclonal antibody (Proteintech, 1:200, CL488‐66828); CoraLite 647‐conjugated angiopoietin‐2 polyclonal antibody (Proteintech, 1:200, 24613‐1‐AP); CoraLite Plus 488‐conjugated collagen type I monoclonal antibody (Proteintech, 1:200, CL488‐67288); and CoraLite 647‐conjugated PDGF‐BB recombinant monoclonal antibody (Proteintech, 1:200, 83214‐5‐RR). Among these, BMP2, osteocalcin/OCN, VEGFA, angiopoietin‐2, and PDGF‐BB were directly labeled with fluorophores using FlexAble Antibody Labeling Kits (Proteintech; KFA501, KFA521, KFA509, KFA529, KFA523).

### 5.25 Animal Ethics Statement

All animal experiments were reviewed and approved by the Experimental Animal Welfare and Ethics Committee of the Laboratory Animal Center, Ningbo University (approval number: **NBU20250390**). All procedures involving animals were performed in accordance with the institutional guidelines for animal care and use and complied with relevant national and local regulations governing laboratory animal welfare. Every effort was made to minimize animal suffering, reduce the number of animals used, and employ humane endpoints whenever appropriate.

## Author Contributions

**Renhao Ni**: investigation, validation, visualization, writing – original draft, writing – review and editing, software, data curation, resources, conceptualization. **Xinyi Shi**: investigation, validation, software, formal analysis, supervision, data curation, writing – original draft. **Tong Zhu**: conceptualization, methodology, validation, supervision, data curation, software. **Guanrong Li**: conceptualization, investigation, validation, formal analysis. **Lei Chang**: investigation, writing – original draft, writing – review and editing, methodology, software. **Shengtao Li**: conceptualization, investigation, validation, formal analysis. **Jingyi Chi**: conceptualization, investigation, software, supervision, methodology. **Chen Shi**: conceptualization, investigation, validation, visualization. **Zeming Hu**: conceptualization, investigation, validation, methodology. **Rong Xu**: conceptualization, investigation, validation, formal analysis. **Yingchun Zhu**: conceptualization, investigation, validation, project administration. **Yang Luo**: conceptualization, investigation, funding acquisition, project administration, writing – review and editing. **Yichen Chen**: writing – review and editing, conceptualization, investigation, visualization, project administration. **Fan Yang**: conceptualization, writing – review and editing, funding acquisition, project administration, resources. **Yabin Zhu**: funding acquisition, writing – original draft, writing – review and editing, project administration, resources, formal analysis.

## Funding

This work was supported by the National Natural Science Foundation of China (Grant No. 62574131), the Fundamental Research Funds for the Central Universities (Grant No. YG2025ZD18), the Shanghai Municipal Health Commission (Grant No. 2024ZZ2002), the Innovative Research Team of High‐level Local Universities in Shanghai, the Natural Science Foundation of Zhejiang Province (Grant No. LKLY25H180012), the Major Project of 2035 Sci&Tech Innovation of Ningbo (Grant Nos. 2024Z034 and 2024Z204), the Ningbo Top Medical and Health Research Program (Grant No. 2024021020).

## Conflicts of Interest

The authors declare no conflicts of interest.

## Supporting information




**Supporting File**: advs78616‐sup‐0001‐SuppMat.docx.

**Supporting video**: advs78616‐sup‐0002‐VideoS1.mp4.

**Supporting video**: advs78616‐sup‐0002‐VideoS2.mp4.

**Supporting video**: advs78616‐sup‐0002‐VideoS3.mp4.

**Supporting video**: advs78616‐sup‐0002‐VideoS4.mp4.

**Supporting video**: advs78616‐sup‐0002‐VideoS5.mp4.

## Data Availability

The data that support the findings of this study are available on request from the corresponding author. The data are not publicly available due to privacy or ethical restrictions.
