# Supplementary material for: Development‐Informed Microsphere Assemblies Enable Spatiotemporally Programmed Bone–Vascular Organoid‐Like Constructs for Large Bone Defect Repair
Source: Adv Sci (Weinh). 2026 Nov 9:e78616. Online ahead of print. doi: 10.1002/advs.78616 (PMC13748999; doi:10.1002/advs.78616)
Supplement: Supplementary file 1 [file ADVS-9999-e78616-s003.docx]

**Supplementary Information**

**Development-Informed Microsphere Assemblies Enable Spatiotemporally Programmed Bone–Vascular Organoid-Like Constructs for Large Bone Defect Repair**

Renhao Ni^a,b†^, Xinyi Shi^a†^, Tong Zhu^a,f†^, Guanrong Li^a,f^, Lei Chang^b^, Shengtao Li^c,d^, Jingyi Chi^a^, Chen Shi^b^, Zeming Hu^a^, Rong Xu^a,f^, Yingchun Zhu^f^, Yang Luo^f^*, Yichen Chen^e^*, Fan Yang^b^*, Yabin Zhu^a^*

^a^ Health Science Center, Ningbo University, Ningbo 315211, China

^b^ Department of Orthopaedics, Shanghai Key Laboratory for Prevention and Treatment of Bone and Joint Diseases, Shanghai Institute of Traumatology and Orthopaedics, Ruijin Hospital, Shanghai Jiao Tong University School of Medicine, Shanghai, 200025, China

^c^ Chinese Academy of Medical Sciences & Peking Union Medical College, Beijing 100370, China

^d^ State Key Laboratory of Experimental Hematology, National Clinical Research Center for Blood Disease, Haihe Laboratory of Cell Ecosystem, Institute of Hematology & Blood Diseases Hospital, Tianjin 300020, China

^e^ Central Laboratory, Women and Children’s Hospital of Ningbo University, Ningbo, China, Ningbo 315211, China

^f^ The First Affiliated Hospital of Ningbo University, Ningbo 315020, China

*Corresponding author: zhuyabin@nbu.edu.cn; yf12498@sjtu.edu.cn; feyychenyichen@nbu.edu.cn; fyluoyang@nbu.edu.cn.

^†^These authors contributed equally to this work.

**Figure S1. In silico knockout of *BMP4/Bmp4* or *VEGFA/Vegfa* in BMSCs identifies associated gene signatures and perturbed functional programmes across species. a,b**, Top genes associated with virtual knockout of *Bmp4* (a) or *Vegfa* (b) in mouse BMSCs, ranked by Z score. **c**, Gene Ontology (GO) biological process enrichment of genes associated with Bmp4 knockout (red) or Vegfa knockout (blue) in mouse BMSCs. Enrichment is shown as −log10(adjusted P value); representative terms are highlighted, and selected contributing genes are indicated. **d**, GO biological process enrichment of genes associated with *BMP4* knockout (red) or *VEGFA* knockout (blue) in human BMSCs, displayed as in c.

**Figure S2. Swelling and degradation of composite hydrogels over 14 days and** **Characterization of gelatin methacrylation.** (a) ^1H NMR^ spectra of native gelatin and gelatin methacrylate (GelMA). Insets enlarge the low-field region, where GelMA shows new vinyl proton resonances of the methacryloyl group (≈5–6.5 ppm; arrows) that are absent in gelatin, confirming successful functionalization. (b) FTIR spectra of gelatin (black) and GelMA (red). GelMA exhibits additional/shifted bands attributable to methacrylate C=C and =C–H vibrations and a strengthened ester C=O signal relative to amide bands, consistent with introduction of methacryloyl groups. (c) Time-dependent swelling (%) of four formulations: Control (GelMA), BG (GelMA + bioactive glass), PEO (GelMA + PEO), and PEO/BG (GelMA + PEO + bioactive glass). (d) Mass-loss profile (“Degradation”, plotted as percentage of initial dry mass remaining) for the same formulations from Day 0 to Day 14. Error bars indicate mean ± SD of replicates. Rapid early changes (Day 1–3) are followed by near-plateaus; GBG and GPBG show lower swelling and slower mass loss than G and GP. (N=5).

**Figure S3. Isolation and characterization of rabbit bone-marrow mesenchymal stem cells (BMSCs).** (a-c) Trilineage differentiation confirming multipotency after lineage-specific induction: osteogenic (a), adipogenic (b), and chondrogenic (c). (d-i) Immunofluorescent phenotyping under basal culture. BMSCs express the MSC markers CD44 (d, green) and CD90 (g, green), while hematopoietic/monocytic markers CD34 (e, red) and CD14 (h, red) are absent. Merged images are shown in (f) and (i); nuclei are counterstained with DAPI (blue). (j) Flow cytometry characterization of BMSCs. Cells exhibit high expression of mesenchymal stem cell markers CD90 and CD44, with minimal expression of hematopoietic/monocytic markers CD14 and CD34.

**Figure S4.** **Plasmid maps of the overexpression constructs.** (a–b) Plasmid maps of the overexpression constructs: BMP4 (a) and VEGFA (b) in pCMV-3×FLAG-Neo backbones.

**Figure S5. Three-dimensional fluorescence evaluation of osteo-induction by Bone Organoids constructed under different conditions. a, b,** Representative 3D immunofluorescence images of (a) BMP2 and (b) osteocalcin (OCN) within aMS at day 3 and day 5 following four treatments: BMSC (PEO aMS seeded with BMSCs, no stimulus), BMSC^BMP4^ (PEO aMS seeded with BMP4-primed BMSCs; paracrine only), BMSC/BG (PEO/BG aMS seeded with BMSCs; ionic release only) and BMSC^BMP4^/BG (PEO/BG aMS seeded with BMP4-primed BMSCs; combined paracrine + ionic release). Green, BMP2 or OCN; red, F-actin; blue, nuclei. Scale bars, 200 µm (all images). **c, d,** Mean fluorescence intensity (MFI) of (c) BMP2 and (d) OCN within 3D microspheres for the four groups (n = 3 independent samples). Data are presented as mean ± s.d. Asterisks denote statistical significance for the indicated pairwise comparisons among the experimental groups; number signs denote comparisons between BMSC and BMSC^BMP4^/BG. ***p*** < 0.05, ***p*** < 0.01, ***p*** < 0.001; ##***p*** < 0.01, ###***p*** < 0.001.

**Figure S6. Original, uncropped Western blot images.** Note: Given the similar molecular weights of BMP4, WNT5A, and β-actin, the membrane was first probed and imaged for BMP4 and WNT3A, stripped with antibody stripping buffer to remove bound antibodies, and then reprobed with anti-β-actin for a second exposure.

**Figure S7 Principal-component analysis (PCA) of global transcriptomes.** (a) That shows clear segregation among Blank (N, native BMSC microspheres), BMP4-overexpressing BMSC microspheres (B), 1393 bioactive-glass microspheres (G) and the cooperative group (BG); PC1 + PC2 explain 97.9 % of total variance. (b) KEGG pathway enrichment analysis of genes significantly up-regulated in BMSC^BMP4^/BG versus Blank BMSC microspheres highlights pathways related to ECM–receptor interaction, focal adhesion, cytokine–cytokine receptor interaction, Wnt signaling, TGF-β signaling, Hippo signaling, PI3K–Akt signaling, mTOR signaling, glycosaminoglycan metabolism and MAPK signaling. Dot size reflects the number of genes per pathway, and colour indicates the adjusted p value. (c) Chord diagram showing representative up-regulated genes in BMSC^BMP4^/BG (outer gene labels) and their connection to key enriched pathways (outer coloured sectors). Ribbon colours correspond to KEGG pathways as indicated in the legend, and the colour scale denotes the log fold change (logFC) of each gene.

**Figure S8. Conditioned medium from BMP4/VEGFA-engineered BMSCs promotes VEC migration in a concentration- and time-dependent manner.** (a) Representative images of crystal violet-stained VECs that migrated through Transwell membranes after 24 or 48 h of incubation. VECs were seeded in the upper chambers, while the lower chambers contained basal medium (Ctrl) or conditioned medium collected from BMP4/VEGFA-engineered BMSCs (BMSC^BMP4+VEGFA^-CM) diluted to 10%, 25%, or 50% (v/v) with basal medium. After incubation, non-migrated cells remaining on the upper surface of the membrane were removed, whereas cells that had migrated to the lower surface were fixed and stained with crystal violet. Scale bar, 200 μm. (b) Quantification of migrated VECs under the indicated conditions. Migrated cells were counted in five randomly selected microscopic fields per Transwell insert, and the mean value of the five fields was treated as one independent sample. Data are presented as the mean ± SD from three independent experiments. Statistical significance was determined using one-way ANOVA followed by Tukey’s multiple-comparisons test. ###p < 0.001 versus the corresponding Ctrl group; **p* < 0.05 and ***p* < 0.01 for the indicated comparisons.

**Figure S9. Timeline and schematic illustration of the rabbit distal femoral defect experiment.** After a 7-day acclimatization period, New Zealand White rabbits were randomly assigned to the Sham, Defect, and bone–vascular OLCs groups. The Sham group underwent surgical exposure without bone drilling, the Defect group received a cylindrical distal femoral defect measuring 5 mm in diameter and 7 mm in depth, and the bone–vascular OLCs group received the same defect followed by immediate implantation of seven-day-matured bone–vascular OLCs. Femoral specimens were harvested at 4 and 8 weeks after surgery for micro-CT and histological analyses. Six animals from each group were analyzed at each time point.

**Figure S10 Histopathology of major organs after bone–vascular OLCs implantation shows no systemic immune rejection.** Hematoxylin–eosin (H&E) staining of heart, liver, spleen, lungs, and kidney harvested at 4 weeks and 8 weeks from New Zealand White rabbits receiving either Defect (sham/defect only) or bone–vascular OLCs (implanted OLCs/microsphere construct). Across all time points and organs, tissue architecture remains preserved: myocardium arranged regularly without myocytolysis; hepatic lobular structure intact without portal or lobular inflammatory infiltrates; splenic white/red pulp organization maintained without granulomatous lesions; alveolar spaces open without edema, hemorrhage, or leukocytic exudates; and renal glomeruli/tubules intact without interstitial nephritis or fibrosis. No necrosis, microthrombi, or foreign-body–type granulomas are observed in distant organs, indicating no evident systemic toxicity or immune rejection associated with implantation. H&E; representative images from n=3 animals per group and time point. Scale bars, 200 μm.

**Figure S11. Independent and combined effects of BMP4 and VEGFA modulation on osteogenic- and vascular-associated marker expression in bone–vascular OLCs.** (a) Representative immunofluorescence images of bone–vascular OLCs generated from BMSCs subjected to different combinations of BMP4 and VEGFA knockdown (KD) or overexpression (OE). The four groups were BMP4 KD/VEGFA KD, BMP4 OE/VEGFA KD, BMP4 KD/VEGFA OE, and BMP4 OE/VEGFA OE. BMP2, an osteogenesis-associated marker, is shown in green; PDGFB, a vascular-associated signaling marker, is shown in yellow; F-actin was stained with phalloidin in red; and nuclei were counterstained with DAPI in blue. Scale bars, 100 μm. (b,c) Quantification of the relative mean fluorescence intensities of BMP2 (b) and PDGFB (c). Fluorescence intensities were normalized to those of the BMP4 KD/VEGFA KD group. Data are presented as the mean ± SD from three independent biological replicates. Statistical significance was determined using one-way ANOVA followed by Tukey’s multiple-comparisons test. ns, not significant; **p* < 0.05; ****p* < 0.001.

**Figure S12. Assessment of cell viability and inflammatory response following *BMP4* and *VEGFA* overexpression in BMSCs.** (a) Flow cytometry analysis of apoptosis in BMSCs transfected with BMP4, VEGFA, or BMP4+VEGFA plasmids. Cells were stained with Annexin V-FITC and PI. Quadrants Q2+Q3 indicate apoptotic cell populations. (b–c) Relative mRNA expression levels of inflammatory cytokines TNF-α (b) and IL-1β (c) measured by qRT-PCR in transfected BMSCs. Data are presented as mean ± SD (n = 3), and statistical analysis was performed using one-way ANOVA; ns indicates no significant difference.

**Figure S13. Integrated hdWGCNA, CellChat, and NicheNet analyses identify BMP4 and VEGFA as candidate BMSC-derived ligands.** (A) Gene dendrogram of co-expression modules identified by hdWGCNA. (B) Heatmap showing the correlations between hdWGCNA modules and the major cell types. (C) UMAP visualization of hdWGCNA modules, showing the distribution of genes assigned to each module, with BMP4 and VEGFA highlighted. (D) Distribution of hdWGCNA module eigengene activity across the t-SNE embedding. (E) CellChat circle plots showing the number and strength of inferred intercellular communications among the major cell populations. (F,G) UpSet plots showing the intersections among BMSC differentially expressed genes, genes within the hdWGCNA blue module, and function-specific Gene Ontology gene sets, including bone mineralization (F) and endothelial cell differentiation (G). CellChat, NicheNet, and integrated prioritization scores of the intersecting candidate genes are also shown.

**Figure S14. siRNA-mediated knockdown of BMP4 and VEGFA suppresses the expression of their** **osteogenesis- and angiogenesis-associated genes in BMSCs.** (a,b) Heatmaps showing the relative expression profiles of genes associated with BMP4-mediated osteogenic signaling (a) and VEGFA-mediated angiogenic signaling (b) in BMSCs transfected with negative control siRNA (NC), siBMP4, or siVEGFA. Each column represents an independent biological replicate, and the color scale indicates row-wise standardized expression levels (Z-scores). (c–g) qRT-PCR analysis of the mRNA expression levels of ***BMP4*** (c), ***BMP2*** (d), ***COL1A1*** (e), ***RUNX2*** (f), and ***SMAD5*** (g) following BMP4 knockdown. (h–k) qRT-PCR analysis of the mRNA expression levels of ***VEGFA*** (h), ***FGFR2*** (i), ***KDR*** (j), and ***VCAM1*** (k) following *VEGFA* knockdown. Gene expression levels were normalized to those of the corresponding NC group. Data are presented as the mean ± SD of three independent biological replicates. Statistical significance was determined using an unpaired two-tailed Student’s *t*-test. ***p* < 0.01, ****p* < 0.001.

**Figure S15. Reconstructed images are presented from different XY viewing orientations to visualize the spatial distribution of cells and vascular-associated markers throughout the constructs.** (a,b) Whole-mount confocal z-stack imaging and three-dimensional reconstruction of bone–vascular organoid-like constructs after 72 h of culture in the BMSC control group (a) and BMSC^BMP4+VEGFA^ group (same sample) (b). Nuclei were stained with DAPI (blue), CD31 was labeled in green, VEGFA in magenta, and F-actin with phalloidin (red). Single-channel images are shown on the left, merged whole-mount reconstructions in the center, and enlarged views of the boxed regions on the right. Scale bars: 500 μm for whole-mount images and 100 μm for enlarged images.

**Table S1** Primers used for RT-qPCR

| **Gene** | **Forward sequence** | **Reverse sequence** |
| --- | --- | --- |
| ***BMP4*** | GGAGCTTCCACCACGAAGAA | GGAAGCCCCTTTCCCAATCA |
| ***RUNX2*** | TCGGAGAGGTACCAGATGGG | CATTCCGGAGCTCAGCAGAA |
| ***COL1A1*** | AGTGGTTTGGATGGTGCCAA | GCACCATCATTTCCACGAGC |
| ***BMP2*** | CGAAATTCCCCGTGACCAGA | TGTTTCTCCTCCAAGTGGGC |
| ***SMAD5*** | TGTTGGGCTGGAAACAAGGT | CCTGCAGGCGTCCATCTAAA |
| ***VEGFA*** | CACCGAGGAGTTCAACGTCA | TTTCGTTTTTGCCCCTTGCC |
| ***FGFR2*** | GACCAAACGTATCCCCCTGC | TGTCTGCCGTTGAAGAGAGG |
| ***VCAM1*** | CGAATGAGGGGACCACATCT | GACTGTGATCGGCTTCCCAG |
| ***KDR*** | TACACCTGTGCAGCATCCAG | CGTGGCTTCCACCAGAGATT |
| ***TNF-α*** | TCAGAGGGCCTGTACCTCAT | GGAGGTTGACCTTGGTCTGG |
| ***IL-1β*** | CCAGCTACGAATCTCCGACC | CATGGCCACAACAACTGACG |
| ***TGF-β1*** | TGATGTCACCGGAGTTGTGC | GTGAACCCGTTGATGTCCACT |
| ***Ki-67*** | TATCAAAAGGAGCGGGGTCG | ACCCCTTCCAAACAAGCAGG |
| ***ANGPT2*** | TTGGCCGCAGCCTATAACAA | ACAGCATTGGACACGTAGGG |
| ***GAPDH*** | CCACTTTGTGAAGCTCATTTCCT | TCGTCCTCCTCTGGTGCTCT |

**Table S2** SiRNA Sequence

| **Gene** | **SiRNA** | **Sense strand (5′–3′)** | **Antisense strand (5′–3′)** |
| --- | --- | --- | --- |
| ***BMP4*** | **si-BMP4-17** | GAAUGCUGAUGGUCGUUUUdTdT | AAAACGACCAUCAGCAUUCdTdT |
| ***BMP4*** | **si-BMP4-27** | GGUCGUUUUAUUAUGCCAAdTdT | UUGGCAUAAUAAAACGACCdTdT |
| ***BMP4*** | **si-BMP4-63** | GAGCCAUGCUAGUUUGAUAdTdT | UAUCAAACUAGCAUGGCUCdTdT |
| ***VEGFA*** | **si-VEGFA-743** | AGAUCGAGUACAUCUUCAAdTdT | UUGAAGAUGUACUCGAUCUdTdT |
| ***VEGFA*** | **si-VEGFA-852** | GCAGAUUAUGCGGAUCAAAdTdT | UUUGAUCCGCAUAAUCUGCdTdT |
| ***VEGFA*** | **si-VEGFA-1003** | CGCAAGAAAUCCCGGUAUAdTdT | UAUACCGGGAUUUCUUGCGdTdT |
